# Impact of Life Stressors on Myalgic Encephalomyelitis/Chronic Fatigue Syndrome Symptoms: An Australian Longitudinal Study

**DOI:** 10.3390/ijerph182010614

**Published:** 2021-10-11

**Authors:** Cassandra Balinas, Natalie Eaton-Fitch, Rebekah Maksoud, Donald Staines, Sonya Marshall-Gradisnik

**Affiliations:** 1 The National Centre for Neuroimmunology and Emerging Diseases, Menzies Health Institute Queensland, Griffith University, Gold Coast 4215, Australia; c.balinas@griffith.edu.au (C.B.); natalie.eaton-fitch@griffithuni.edu.au (N.E.-F.); d.staines@griffith.edu.au (D.S.); s.marshall-gradisnik@griffith.edu.au (S.M.-G.); 2 Consortium Health International for Myalgic Encephalomyelitis, Menzies Health Institute Queensland, Griffith University, Gold Coast 4215, Australia; 3 School of Medical Science, Griffith University, Gold Coast 4215, Australia

**Keywords:** Myalgic Encephalomyelitis, Chronic Fatigue Syndrome, life stressor, longitudinal, work, income, family

## Abstract

(1) Background: Myalgic Encephalomyelitis/Chronic Fatigue Syndrome (ME/CFS) is a complex, multifaceted illness. The pathomechanism, severity and progression of this illness is still being investigated. Stressors have been implicated in symptom exacerbation for ME/CFS, however, there is limited information for an Australian ME/CFS cohort. The aim of this study was to assess the potential effect of life stressors including changes in work, income, or family scenario on symptom severity in an Australian ME/CFS cohort over five months; (2) Methods: Australian residents with ME/CFS responded to questions relating to work, income, living arrangement, access to healthcare and support services as well as symptoms experienced; (3) Results: thirty-six ME/CFS patients (age: 41.25 ± 12.14) completed all questionnaires (response rate 83.7%). Muscle pain and weakness, orthostatic intolerance and intolerance to extreme temperatures were experienced and fluctuated over time. Sleep disturbances were likely to present as severe. Work and household income were associated with worsened cognitive, gastrointestinal, body pain and sleep symptoms. Increased access to healthcare services was associated with improved symptom presentation; (4) Conclusions: life stressors such as work and financial disruptions may significantly contribute to exacerbation of ME/CFS symptoms. Access to support services correlates with lower symptom scores.

## 1. Introduction

Myalgic Encephalomyelitis/Chronic Fatigue Syndrome (ME/CFS) is a complex and disabling condition that affects an estimated 200,000 Australians [1] with a substantial economic burden estimated at $14.5 billion (AUD) in Australia per annum [2]. The pathomechanism underlying ME/CFS is not fully understood. Diagnosis is heavily dependent on the application of case criteria including the Fukuda criteria (1994), the Canadian Consensus Criteria (CCC), and the International Consensus Criteria (ICC) following the exclusion of any other pathological cause [3,4,5]. Under these criteria, ME/CFS is hallmarked by post-exertional neuroimmune exhaustion (PENE). PENE presents as profound fatigue following minimal exertion, is unrelieved by rest, and exacerbates other symptoms that affect multiple body systems [3,4]. These symptoms include immune dysfunction, gastrointestinal, genitourinary, cardiovascular, and neurological issues [4]. The severity of these symptoms varies between patients ranging from mild to severe, where approximately 25% of patients are either housebound or bedbound [4]. Many patients are dependent on carer support and require access to numerous multidisciplinary services including various specialists, physio- and occupational therapy [6,7]. Currently there is no targeted treatment or molecular-based diagnostic test for this illness and existing treatment guidelines attempt to relieve specific symptoms [8].

The impact of stress on chronic illnesses has been described in various maladies and chronic illnesses including anxiety/depression, cancer, and heart and lung disease [9]. In an Australian epidemiological investigation, almost half of ME/CFS patients whose ME/CFS onset was attributed to a non-infectious trigger listed ‘undue stress’ as a significant contribution to illness onset [10]. A study conducted by Lutgendorf et al. in 1995 suggested that physical symptoms of ME/CFS were exacerbated by stress from Hurricane Andrew [11]. Chu et al. acknowledged stress as a potential trigger of flare- ups and symptom aggravation. Importantly, greater stress management is associated with reduced symptom severity and the improvement of the illness burden [12].

This current and novel investigation aimed to determine the impact of life stressors and access to professional or familial support on symptom severity presentation. This investigation is the first pilot study to investigate the contribution of these life stressors longitudinally and in Australian ME/CFS patients.

## 2. Materials and Methods

### 2.1. The Study Design and Recruitment

This study implements a longitudinal design with data collected over a five- month period from April 2020 to January 2021. A total of 43 ME/CFS patients responded to online recruitment advertisements and were enrolled in this project. To be eligible to participate in this study, participants had to (1) meet the Fukuda, CCC or ICC criteria and be diagnosed with ME/CFS by a healthcare practitioner; (2) be between 18 and 65 years of age; and (3) be an Australian resident. Of the 43 participants who enrolled, seven only completed the surveys in part and were therefore withdrawn from the study, leaving 36 participants who successfully completed questionnaires over the five-month period (response rate: 83.7%).

This study was approved by the Griffith University Human Research Ethics Committee (HREC) (project ID: 2016/502) and Gold Coast University Hospital HREC (project ID: 56469). Participants provided written informed consent through reading and agreeing to terms and conditions of the study at the beginning of the entry questionnaire. Participants were anonymised with an alphanumeric code.

### 2.2. Data Collection

All participants were required to complete an online questionnaire to gather sociodemographic background, medical history, and symptom presentation. This questionnaire utilised components of the Fukuda, CCC and ICC criteria as well as the Australian economic survey (2020) and was administered via an online software, LimeSurvey (Carsten, Schmitz, Hamburg, Germany) [2]. Participants were excluded if they reported a history of alcohol abuse, diabetes mellitus, malignancies, or autoimmune disease. Participants were then invited to complete a series of questionnaires which were composed of questions detailing experiences of stress and/or change in work, loss of income or change in family dynamic. The survey was completed by participants every two weeks, for a total of five months. Participants completed the survey every two weeks to allow better recall, especially as brain fog and cognitive impairment is commonly experienced in ME/CFS, however, only monthly data was analysed.

### 2.3. Sociodemographic Characteristics

The following sociodemographic data was collected cross-sectionally via the eligibility questionnaire: (1) age, (2) age of onset, (3) gender [male, female, or other], (4) BMI (kg/m^2^), (5) location (by Australian state or territory), (6) current employment [unemployed, casual, part-time, full-time], and (7) education. Body mass index (BMI) was categorised according to the World Health Organization (WHO): underweight (<18.5), normal weight (18.5 to 24.9), overweight (25.0 to 29.9), and obese (≥30.0). Education was categorised according to highest level completed [primary school, high school, professional training, undergraduate or postgraduate/doctoral].

### 2.4. Lifestyle Factors

The first section of the questionnaire requests background information on lifestyle factors for that particular time period. These questions include: (1) hours worked [N/A, 1–15 h/week, 16–24 h per week, 25–34 h/week or ≥ 35 h/week], (2) whether there was a change in hours [yes/no], (3) whether the income is sustainable for household needs [yes/no], (4) change in household income [yes/no], (5) current living arrangement [married, single, divorced/separated, window/er, single with children, married/ de facto with children].

### 2.5. Access to Healthcare and Support

ME/CFS patients were surveyed about their access to healthcare services. Healthcare services included: (1) general practitioner, (2) nurse, (3) pathologist, (4) medical specialist, (5) occupational therapist, (6) physiotherapist (v7) other. The subsequent component of this questionnaire assessed patient access to professional and unpaid support including (1) childminding/ day-care, (2) household chores, (3) personal support, (4) meals/ grocery shopping, (5) transport, (6) other. Frequency across the fortnight was measured using the following four-point scale: 0, 1–2 times, 3–4 times and >5 times per fortnight.

### 2.6. Symptom Severity

The participants were requested to rate their experience of a variety of symptoms. These symptoms related to nine different domains derived from the Fukuda, CCC and ICC criteria: (1) cognitive, (2) pain, (3) sleep disturbances, (4) sensory sensitivity, (5) infection or flu- like symptoms, (6) gastrointestinal symptoms, (7) orthostatic issues, (8) impaired temperature regulation (9) other. Symptom severity was measured using a five-point scale: none, mild, moderate, severe, and extreme.

### 2.7. Statistical Analysis

Statistical analysis was conducted using IBM SPSS Statistics 27 software package. Descriptive statistics were calculated and presented as n (%). Normality was assessed using the Shapiro-Wilk test. The Friedman test was conducted to determine differences within each ordinal parameter and the longitudinal effect on these parameters. Significant outputs were followed up with Wilcoxon Signe-ranked post-hoc tests. Wilcoxon Signe-ranked post-hoc tests were selected as the data compared are related. Partial correlations accounting for cofactors including gender, age, education, and employment were calculated. The significance for both statistical tests were measured at a 95% confidence interval represented by *p* < 0.05.

## 3. Results

### 3.1. Sociodemographic Characteristics

Sociodemographic characteristics are presented in Table S1. The study period spanned from April 2020 to March 2021. All participants were diagnosed according to the CCC definition. The average age of the participants was 41.25 ± 12.14. The average onset age was 27.60 ± 11.32. A greater proportion of the participants were female (63.9%). Participants were located across most states and territories of Australia excluding the Northern Territory and Tasmania. The greatest proportion of participants resided in Queensland (58.3%), had a normal BMI (58.3%), and were unemployed (50.0%).

### 3.2. Family, Financial, and Work-Related Parameters

Table S2 summarises the frequencies of patient responses of various family, financial and work- related parameters. An association between access to professional and unpaid services positively correlated to symptom scores; the majority did not experience a change in weekly hours worked (~85.56%) and 44.4% of patients were married. There were no changes in living arrangements throughout the study, 58.3% of patients had a sustainable income and 79.44% did not experience a change in household income. Comparison tests showed significant changes in weekly hours worked during various months (Tables S7 and S8, Figure S1). Change in weekly work hours correlated with sustainability of household income (r = 0.253, *p* < 0.001) and a change in household income (r = 0.356, *p* < 0.001) (Table S9).

### 3.3. Access to Healthcare, Professional and Unpaid Services

Access to healthcare frequency is presented in Table S3. The most frequently and consistently accessed healthcare services by ME/CFS patients for the duration of the study were general practitioners (GPs). This service was accessed one to two times within the two-week period by approximately 57.78% of patients. Other services that were accessed include nutritionists, radiologists, psychologists, chiropractors, optometrist, and dentists. ME/CFS patients more frequently relied on volunteer help from family and friends in contrast to professional services. The most frequent service that patients needed assistance completing was household chores, followed by meal preparation or grocery shopping (Tables S4 and S5). Accessing professional services that assist with household chores and personal support were highly associated (r = 0.819, *p* < 0.001). Living arrangements also related to required access to paid and unpaid childcare (*p* < 0.05).

### 3.4. Symptom Presentation

Frequency of symptoms experienced are presented in Table S6. Sleep disturbances were the most severe and extreme symptom experienced by patients (32.8% and 12.8% respectively). The most reported moderately experienced symptom was muscle pain (48.36%), followed by muscle weakness (40.54%). Light sensitivity was the most frequent mildly experienced symptom (47.76%). Severity of orthostatic intolerance and intolerance to extreme temperatures significantly changed throughout the duration of the study (*p* < 0.05). Changes in symptom presentation were observed following post-hoc analysis using the Wilcoxon signed-rank test. Specifically, self-reported mild, moderate, and severe neurocognition symptoms (memory and concentration) increased over the period of five months, meanwhile symptoms of pain (headache, muscle pain and multi-joint pain) improved. Complaints of severe sleep disturbances also increased over time. Mild and moderate complaints of flu-like symptoms (sore throat and tender lymph nodes) fluctuated over various time points (Figure S1). Complaints of moderate genitourinary symptoms improved over time compared with baseline. Orthostatic complaints worsened over time, while intolerances to extreme temperatures improved after three and four months.

There were numerous associations found between symptoms experienced. All clinical symptoms primarily reported low to moderate correlations (Table S9). Associations between symptoms and access to services and family, financial and work-related parameters showed either a weak or moderate strength 0.25 < r < 0.75. Low to moderate negative correlations were identified between financial and work-related parameters and various clinical symptoms, including cognitive, sleep disturbance, gastrointestinal, and muscular and joint related symptoms (*p* < 0.05). In the majority of cases, access to healthcare, professional and unpaid services correlated to improved symptom scores (98.9%) (Table S9).

## 4. Discussion

This current investigation was the first to explore the longitudinal effects of life stressors on the clinical presentation of ME/CFS in Australian patients. Patient information was collected through a health update survey involving questions regarding family, financial and work-related parameters, access to healthcare services, professional and unpaid support, and ME/CFS clinical symptomology over a five-month period.

All ME/CFS patients met the CCC case definition criteria. The CCC case definition was used over the Fukuda case definition as a significant limitation of the latter is that it is considered too broad for a definitive diagnosis. Consistent with previous literature, participants in this current investigation were most likely to be in their 40s and female [10,13]. Over half of patients were within the normal BMI range (58.83%). Previous investigations have reported that a high BMI has a significant and negative impact on ME/CFS symptoms and quality of life [14,15].

The economic impact of ME/CFS is represented through the high percentage of participants who reported being unemployed and participants who maintained part-time employment but worked reduced or no hours. However, it is unclear whether work was already disrupted prior to commencing this study. Work disruptions for employed ME/CFS patients was observed over the course of the study, thus changes to sustainability of income were expected. Approximately 41.68% of patients reported an unsustainable income and no significant differences were found over the course of the study. These findings are consistent with a previous systematic review which estimated that approximately 54% of ME/CFS patients are unemployed due to disability and individual income losses approximating at $20,000 annually per household [16,17]. Within all financial and work-related parameters there was low to moderate negative correlations with various clinical symptoms, which is supported by published literature [10].

Collectively, these findings suggest that unsustainable income and unemployment negatively impact the clinical presentation of ME/CFS. This association has been investigated in other conditions, however, this is the first study to examine this relationship longitudinally in ME/CFS patients [18]. Stress has been described as a major driver of disparities in health by the World Health Organization (WHO) [18]. Reduced economic and social productivity can cause stress, anxiety or depression, as well as exacerbate symptoms in individuals suffering chronic conditions, such as ME/CFS [10]. A relationship between unemployment and poorer health has been consistently reported [19,20]. Jain et al. determined that severe cognitive impairment and sleep symptoms were significantly associated and increased with ME/CFS patients with a low household income [19]. Another study also reported that increased muscular, cognitive, neurological, autonomic, and immunological symptoms are consistently associated with unemployment and increased risk of work disability in ME/CFS patients [20]. Interestingly, another study identified that environmental and psychological stressors exacerbate self-reported ME/CFS symptoms following a natural disaster [11]. Given that this study occurred during the COVID-19 pandemic, this unprecedented environmental stressor could have influenced these clinical changes. Stress management in ME/CFS patients has also been related to lower fatigue severity in ME/CFS [12]. While most changes in financial stability were negative, some participants were granted access to financial support packages that may have improved or balanced their financial situation.

Some ME/CFS patients are dependent on caretakers to provide income. Loss of income by their carers may have contributed to the overall change in financial stability. ME/CFS patients require access to multidisciplinary services. Previously published literature has indicated that location and socioeconomic status may influence access to specialist services, particularly in ME/CFS patients [21].

Patients predominantly relied on volunteer help provided by family members or friends for various services including household chores, personal support, grocery shopping, and/ or preparation of meals and transport. Access to unpaid services primarily remained stable with slight fluctuations throughout this investigation. Conversely, professional services were less frequently accessed as more than 80% of patients reported no access to professional services. Access to Australian disability support such as the National Disability Insurance Scheme (NDIS) or pension was not captured in this study in order to understand whether these government initiatives facilitated access to professional care. Some patients reported receiving these funds; however, this data was not specifically collected in this study. ME/CFS patients were not categorised based on severity; however, amongst the cohort of patients included in this study are housebound or bedbound patients that require full-time care. An association between access to professional and unpaid services positively correlated to symptom scores.

The fact that both access to services and the clinical presentation of symptoms correlated with professional and unpaid services may suggest that access to these services by ME/CFS patients may be required to alleviate their burden of illness; however, patients may also limit access to these services if symptoms are stabilized.

### Limitations

As data was collected longitudinally between April 2020 and January 2021, patients’ symptoms could have been influenced by season changes. A study by Bested and Marshall has found that symptom flare-up is precipitated by climate, particularly in winter months [22]. Given that the Health Update survey collected self-reported information, experiences and responses to these changes are highly subjective to each patient, particularly the threshold for symptom severity.

A representative baseline to determine the effects of the COVID-19 pandemic was not feasible as the timing of the pandemic was unexpected. However, collecting this longitudinal information during a period of lockdown and restrictions is still valuable information in terms of capturing any effects on a community that is highly vulnerable and sensitive to external stressors. Comparison with healthy controls during this time would also have been valuable.

Collection of additional data including perceived stress may be useful in allowing inferences to be made on stress effects on ME/CFS symptoms as conducted previously [10,11,12].

## 5. Conclusions

This longitudinal study identified a group of Australian ME/CFS patients who reported significantly reduced levels of full-time employment and financial instability. Access to healthcare and professional and unpaid services were positively correlated to symptom scores, whereas financial and work-related parameters were negatively correlated with symptom scores. Although significant symptom improvements and exacerbations were reported between some months, clinical symptoms fluctuated throughout the study. Symptom fluctuation is a consistent pattern for ME/CFS and may be due to the heterogenous nature of this disease. Importantly, as there were significant negative associations found between stressors such as financial and work-related parameters and symptom scores, this study highlights the importance of stress mitigation and delivery of resources aimed at supporting patients with chronic illnesses who are unable to work, as financial stability may improve symptom experience of ME/CFS. 

## Data Availability

The datasets generated and/or analysed during the writing of this manuscript are not publicly available due to confidentiality agreements, but de-identified data are available from the corresponding author on reasonable request.

## Supplementary Materials

The following are available online at https://www.mdpi.com/article/10.3390/ijerph182010614/s1, Figure S1: Symptom presentation of ME/CFS patients; Table S1: Frequency of Sociodemographic characteristics; Table S2: Frequency of family, financial and work-related parameters; Table S3: Frequency of access to healthcare services; Table S4: Frequency of access to professional services; Table S5: Frequency of access to volunteer services; Table S6: Frequency of symptoms; Table S7: Comparisons of Parameters using Friedman Test; Table S8: Comparisons Wilcoxon Post Hoc Test; Table S9: Partial correlation table.
