# Supplementary material for: Impact of Life Stressors on Myalgic Encephalomyelitis/Chronic Fatigue Syndrome Symptoms: An Australian Longitudinal Study
Source: Int J Environ Res Public Health. 2021 Oct 11;18(20):10614. doi: 10.3390/ijerph182010614 (PMC8535742; doi:10.3390/ijerph182010614)
Supplement: Supplementary file 1 [file ijerph-18-10614-s001.zip › Figure S1.pdf]

**Figure S1**

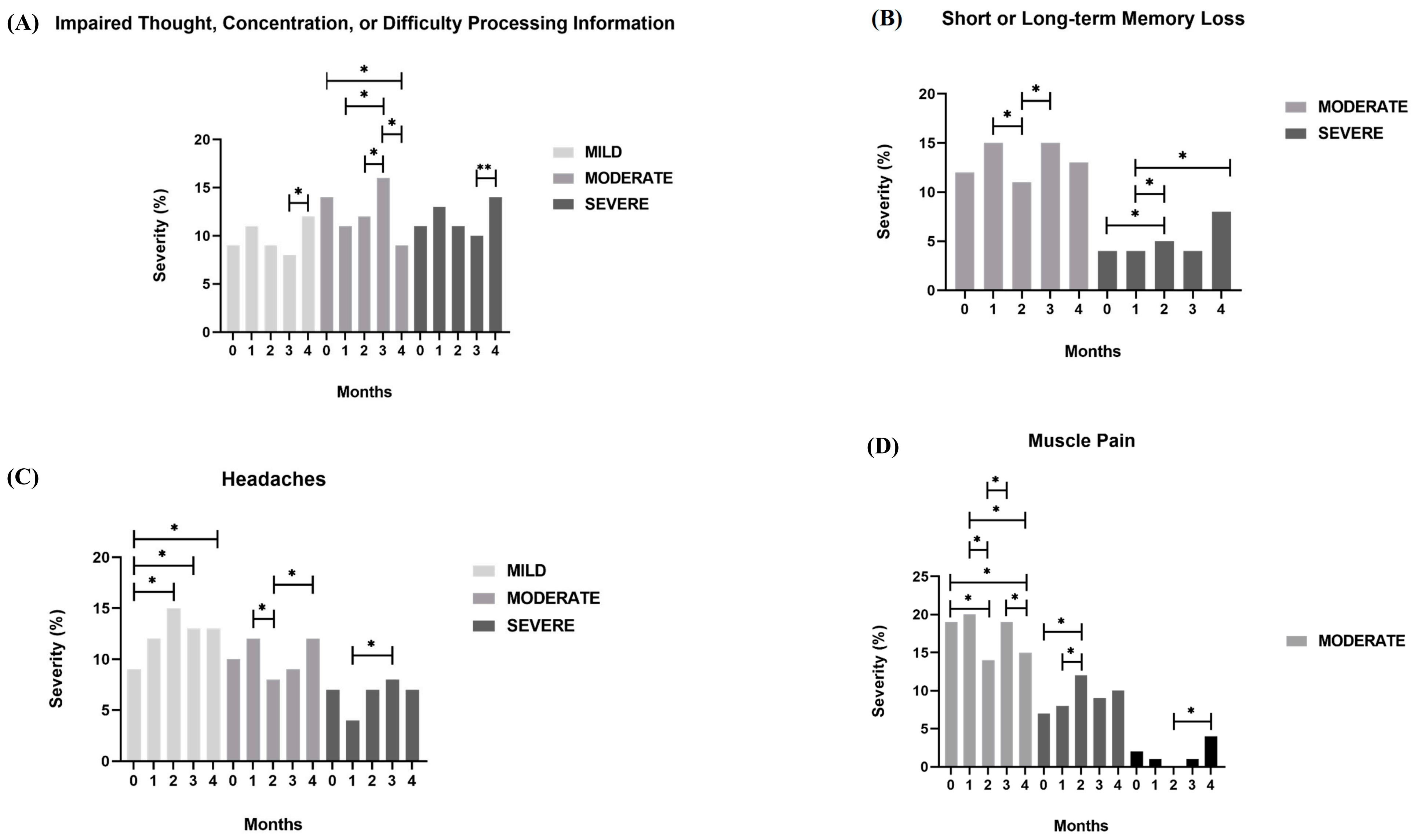
(A) Impaired Thought, Concentration, or Difficulty Processing Information
Severity (%)
Months
MILD
MODERATE
SEVERE
(B) Short or Long-term Memory Loss
Severity (%)
Months
MODERATE
SEVERE
(C) Headaches
Severity (%)
Months
MILD
MODERATE
SEVERE
(D) Muscle Pain
Severity (%)
Months
MODERATE

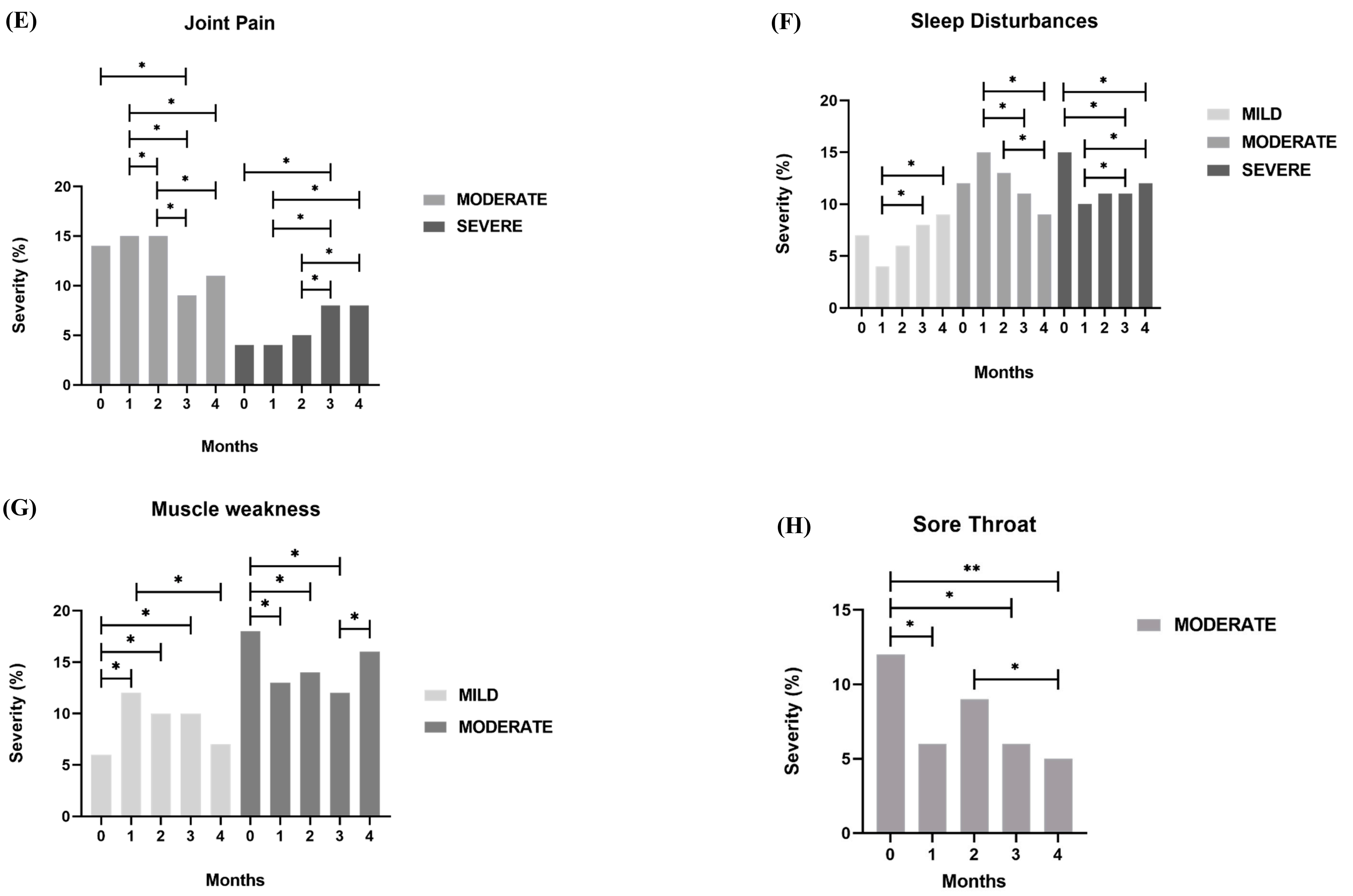
(E)
Joint Pain
Severity (%)
Months
MODERATE
SEVERE
(F)
Sleep Disturbances
Severity (%)
Months
MILD
MODERATE
SEVERE
(G)
Muscle weakness
Severity (%)
Months
MILD
MODERATE
(H)
Sore Throat
Severity (%)
Months
MODERATE

(I)

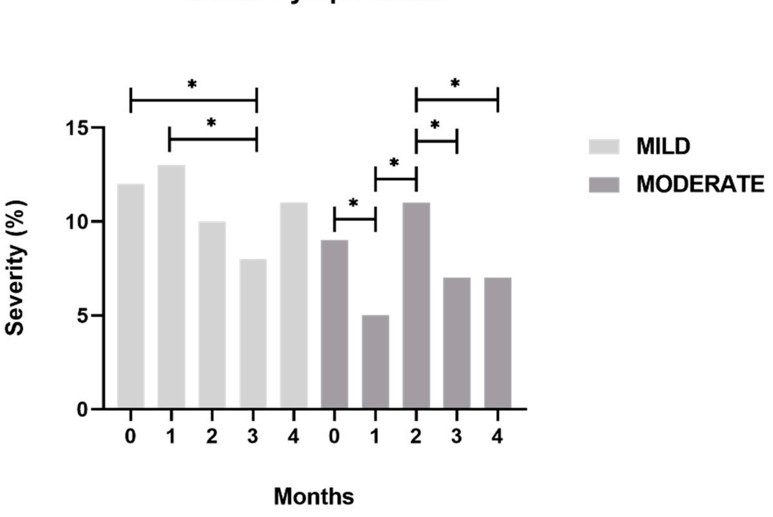
Tender Lymph Nodes
Severity (%)
Months
MILD
MODERATE

(J)

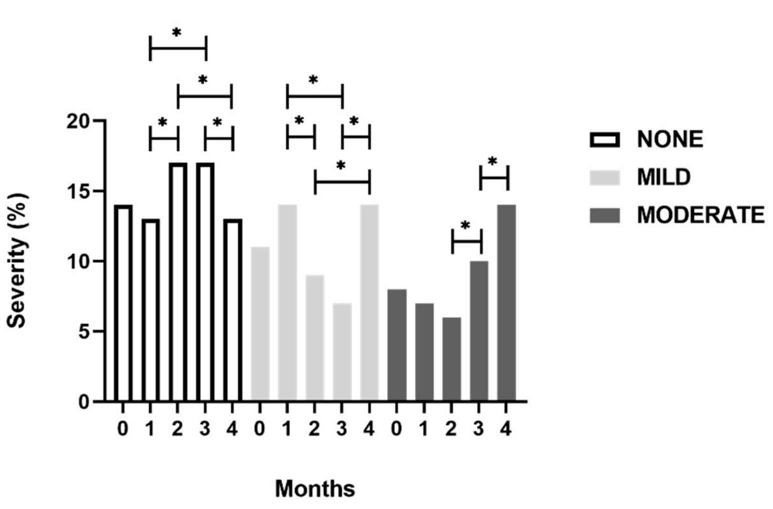
Nausea
Severity (%)
Months
NONE
MILD
MODERATE

(K)

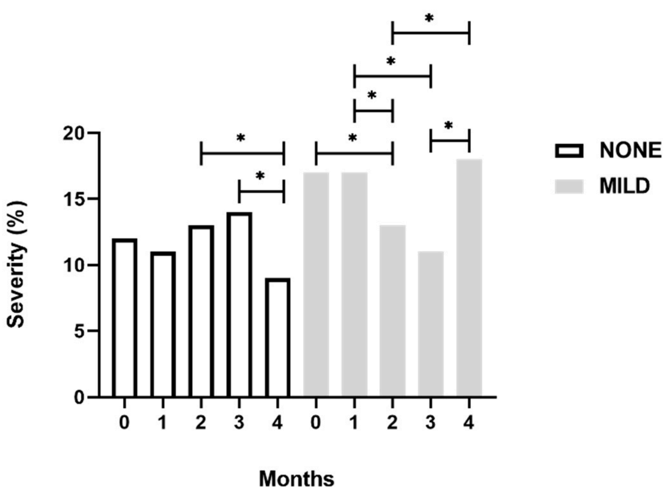
Abdominal Pain
Severity (%)
Months
NONE
MILD

(L)

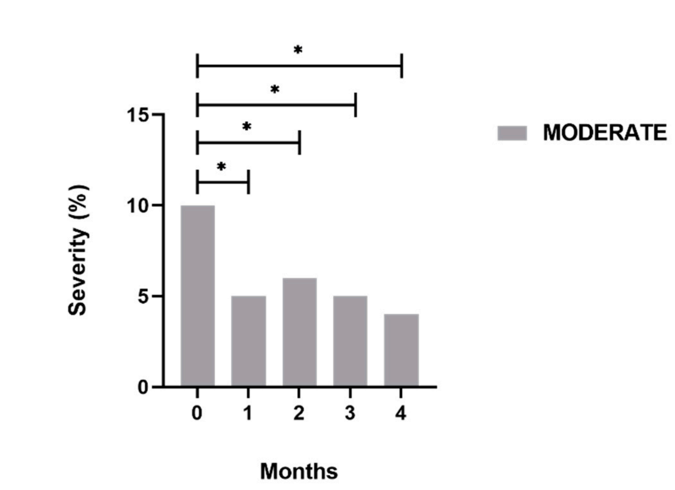
Changes in Frequency and Volume of Urination
Severity (%)
Months
MODERATE

(M) Sensitivity to Food, Medications or Chemicals

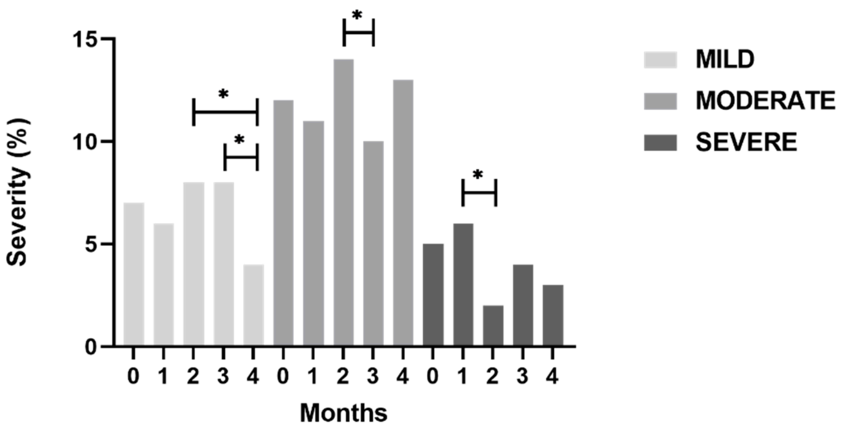


(N) Orthostatic Intolerance (including POTS)

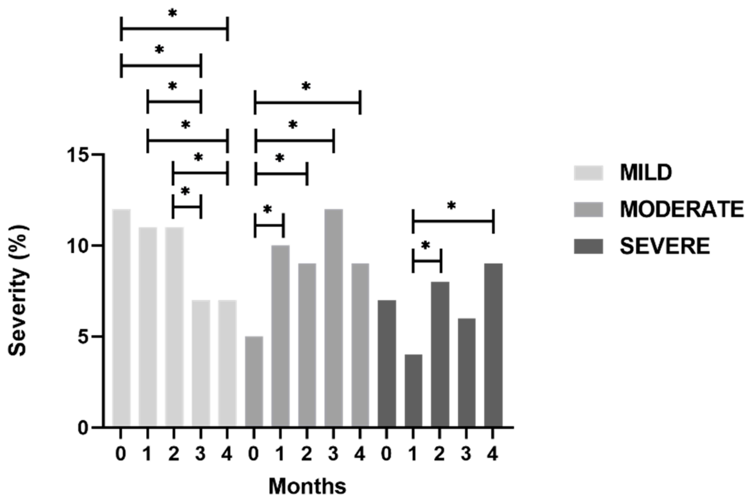


(O) Intolerance to Extreme Temperatures

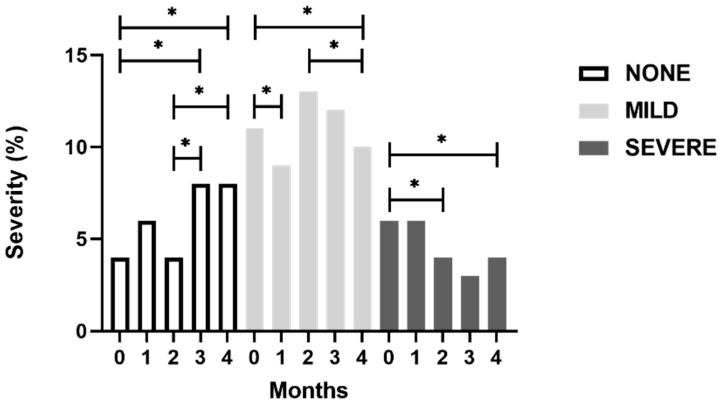

**Fig. S1 Symptom presentation of ME/CFS patients.** Clinical symptoms were categorised into neurological impairment; immune, gastrointestinal, and genitourinary impairments; and energy production/transportation impairments. Symptom severity was measured for five months using a five-point scale (none, mild, moderate, severe, and extreme). Significant differences were measured within each symptom severity and between each month. Only symptom severities that reported significant differences between months are shown. Symptoms included: (A) impaired thought, concentration, or difficulty processing information; (B) short or long-term memory loss; (C) headaches; (D) muscle pain; (E) joint pain; (F) sleep disturbances; (G) muscle weakness; (H) sore throat; (I) tender lymph nodes; (J) nausea; (K) abdominal pain; (L) changes in frequency and volume or urination; (M) sensitivity to food, medications or chemicals; (N) orthostatic intolerance; and (O) intolerance to extreme temperatures. *POTS*, Postural orthostatic tachycardia syndrome. Data are represented as mean ± standard deviation using Wilcoxon Post Hoc tests. Significance is denoted as *($p<0.05$) and ** ($p<0.01$).
