# Supplementary material for: Impact of Life Stressors on Myalgic Encephalomyelitis/Chronic Fatigue Syndrome Symptoms: An Australian Longitudinal Study
Source: Int J Environ Res Public Health. 2021 Oct 11;18(20):10614. doi: 10.3390/ijerph182010614 (PMC8535742; doi:10.3390/ijerph182010614)
Supplement: Supplementary file 1 [file ijerph-18-10614-s001.zip › Table S1. Frequency of Sociodemographic characteristics.pdf]

Table S1. Frequency of Sociodemographic characteristics

	N = 36 (%)
Age (years, mean ± SD)	41.25 ± 12.14
Age of Onset (years, mean ± SD)	27.60 ± 11.32
**Gender**	
Female	23 (63.9%)
Male	13 (36.1%)
Other	0 (0.0%)
**Classification**	
CCC	36 (100%)
**BMI (kg/m^2)**	
Underweight (<18.5)	1 (2.8%)
Normal weight (18.5-24.9)	21 (58.3%)
Overweight (25.0-29.9)	11 (30.6%)
Obese (≥30.0)	3 (8.3%)
**Location**	
New South Wales	2 (5.6%)
Victoria	8 (22.2%)
Queensland	21 (58.3%)
Western Australia	3 (8.3%)
Northern Territory	0 (0.0%)
Australian Capital Territory	1 (2.8%)
Tasmania	0 (0.0%)
South Australia	1 (2.8%)
**Education**	
Primary school	0 (0.0%)
High school	11 (30.6%)
Professional training	11 (30.6%)
Undergraduate	9 (25.0%)
Postgraduate	5 (13.9%)
**Employment**	
Unemployed	18 (50.0%)
Casual	6 (16.7%)
Part-time	11 (30.6%)
Full-time	1 (2.8%)

Abbreviations: BMI, Body Mass Index; CCC, Canadian Consensus Criteria; ICC. International Consensus Criteria.
