# Supplementary material for: Impact of Life Stressors on Myalgic Encephalomyelitis/Chronic Fatigue Syndrome Symptoms: An Australian Longitudinal Study
Source: Int J Environ Res Public Health. 2021 Oct 11;18(20):10614. doi: 10.3390/ijerph182010614 (PMC8535742; doi:10.3390/ijerph182010614)
Supplement: Supplementary file 1 [file ijerph-18-10614-s001.zip › Table S2. Frequency of family, financial and work-related parameters.pdf]

Table S2. Frequency of family, financial and work-related parameters

					N=36 (%)
	**Month 1**	**Month 2**	**Month 3**	**Month 4**	**Month 5**
**Hours per week worked**					
N/A	22 (61.1%)	21 (58.3%)	23 (63.9%)	21 (58.3%)	21 (58.3%)
1-15	5 (13.9%)	6 (16.7%)	13 (36.1%)	7 (19.4%)	6 (16.7%)
16-24	7 (19.4%)	9 (25.0%)	0 (0.0%)	8 (22.2%)	9 (25.0%)
25-34	2 (5.6%)	0 (0.0%)	0 (0.0%)	0 (0.0%)	0 (0.0%)
≥ 35	0 (0.0%)	0 (0.0%)	0 (0.0%)	0 (0.0%)	0 (0.0%)
**Change in weekly hours**					
Yes	9 (25.0%)	4 (11.1%)	4 (11.1%)	5 (13.9%)	4 (11.1%)
No	27 (75.0%)	32 (88.9%)	32 (88.9%)	31 (86.1%)	32 (88.9%)
**Sustainable income**					
Yes	19 (52.8%)	21 (58.3%)	22 (61.1%)	22 (61.1%)	21 (58.3%)
No	17 (47.2%)	15 (41.7%)	14 (38.9%)	14 (38.9%)	15 (41.7%)
**Change in household income**					
Yes	10 (27.8%)	6 (16.7%)	8 (22.2%)	8 (22.2%)	5 (13.9%)
No	26 (72.2%)	30 (83.3%)	28 (77.8%)	28 (77.8%)	31 (86.1%)
**Current living arrangement**					
Married	16 (44.4%)	16 (44.4%)	16 (44.4%)	16 (44.4%)	16 (44.4%)
Single	9 (25.0%)	9 (25.0%)	9 (25.0%)	9 (25.0%)	9 (25.0%)
Divorced/ Separated	2 (5.6%)	2 (5.6%)	2 (5.6%)	2 (5.6%)	2 (5.6%)
Widow/er	0 (0.0%)	0 (0.0%)	0 (0.0%)	0 (0.0%)	0 (0.0%)
Single with children	1 (2.8%)	1 (2.8%)	1 (2.8%)	1 (2.8%)	1 (2.8%)
Married/ De Facto with children	8 (22.2%)	8 (22.2%)	8 (22.2%)	8 (22.2%)	8 (22.2%)
