# Supplementary material for: Impact of Life Stressors on Myalgic Encephalomyelitis/Chronic Fatigue Syndrome Symptoms: An Australian Longitudinal Study
Source: Int J Environ Res Public Health. 2021 Oct 11;18(20):10614. doi: 10.3390/ijerph182010614 (PMC8535742; doi:10.3390/ijerph182010614)
Supplement: Supplementary file 1 [file ijerph-18-10614-s001.zip › Table S3. Frequency of access to healthcare services.pdf]

Table S3. Frequency of access to healthcare services

					N=36 (%)
	**0**	**1**	**2**	**3**	**4**
**GP**					
0	21 (58.3%)	18 (50.0%)	24 (66.7%)	19 (52.8%)	22 (61.1%)
1-2	15 (41.7%)	18 (50.0%)	12 (33.3%)	15 (41.7%)	13 (36.1%)
3-4	0 (0.0%)	0 (0.0%)	0 (0.0%)	2 (5.6%)	1 (2.8%)
>5	0 (0.0%)	0 (0.0%)	0 (0.0%)	0 (0.0%)	0 (0.0%)
**Nurse**					
0	35 (97.2%)	32 (88.9%)	32 (88.9%)	34 (94.4%)	33 (91.7%)
1-2	1 (2.8%)	4 (11.1%)	2 (5.7%)	2 (5.6%)	3 (8.3%)
3-4	0 (0.0%)	0 (0.0%)	1 (2.8%)	0 (0.0%)	0 (0.0%)
>5	0 (0.0%)	0 (0.0%)	0 (0.0%)	0 (0.0%)	0 (0.0%)
**Pathologist**					
0	32 (88.9%)	28 (77.8%)	29 (80.6%)	31 (86.1%)	32 (88.9%)
1-2	4 (11.1%)	8 (22.2%)	6 (16.7%)	4 (11.1%)	3 (8.3%)
3-4	0 (0.0%)	0 (0.0%)	0 (0.0%)	1 (2.8%)	1 (2.8%)
>5	0 (0.0%)	0 (0.0%)	1 (2.8%)	0 (0.0%)	0 (0.0%)
**Medical Specialist**					
0	30 (83.3%)	28 (77.8%)	29 (80.6%)	31 (86.1%)	28 (77.8%)
1-2	4 (11.1%)	7 (19.4%)	6 (16.7%)	4 (11.1%)	7 (19.4%)
3-4	2 (5.6%)	1 (2.8%)	0 (0.0%)	1 (2.8%)	1 (2.8%)
>5	0 (0.0%)	0 (0.0%)	1 (2.8%)	0 (0.0%)	0 (0.0%)
**Occupational Therapist**					
0	33 (91.7%)	35 (97.2%)	35 (97.2%)	33 (91.7%)	31 (86.1%)
1-2	3 (8.3%)	1 (2.8%)	1 (2.8%)	3 (8.3%)	5 (13.9%)

3-4	0 (0.0%)	0 (0.0%)	0 (0.0%)	0 (0.0%)	0 (0.0%)
>5	0 (0.0%)	0 (0.0%)	0 (0.0%)	0 (0.0%)	0 (0.0%)
**Physiotherapist**					
0	33 (91.7%)	32 (88.9%)	33 (91.7%)	28 (77.8%)	31 (86.1%)
1-2	3 (8.3%)	4 (11.1%)	3 (8.3%)	8 (22.2%)	5 (13.9%)
3-4	0 (0.0%)	0 (0.0%)	0 (0.0%)	0 (0.0%)	0 (0.0%)
>5	0 (0.0%)	0 (0.0%)	0 (0.0%)	0 (0.0%)	0 (0.0%)
**Other**					
0	26 (72.2%)	23 (63.9%)	25 (69.4%)	29 (80.6%)	23 (63.9%)
1-2	10 (27.8%)	13 (36.1%)	11 (30.6%)	7 (19.4%)	13 (36.1%)
3-4	0 (0.0%)	0 (0.0%)	0 (0.0%)	0 (0.0%)	0 (0.0%)
>5	0 (0.0%)	0 (0.0%)	0 (0.0%)	0 (0.0%)	0 (0.0%)
