# Supplementary material for: Impact of Life Stressors on Myalgic Encephalomyelitis/Chronic Fatigue Syndrome Symptoms: An Australian Longitudinal Study
Source: Int J Environ Res Public Health. 2021 Oct 11;18(20):10614. doi: 10.3390/ijerph182010614 (PMC8535742; doi:10.3390/ijerph182010614)
Supplement: Supplementary file 1 [file ijerph-18-10614-s001.zip › Table S4. Frequency of access to professional services.pdf]

Table S4. Frequency of access to professional services

					N=36 (%)
	**0**	**1**	**2**	**3**	**4**
**Childminding/ Day-care**					
0	35 (97.2%)	35 (97.2%)	35 (97.2%)	35 (97.2%)	35 (97.2%)
1-2	0 (0.0%)	0 (0.0%)	0 (0.0%)	0 (0.0%)	1 (2.8%)
3-4	1 (2.8%)	1 (2.8%)	1 (2.8%)	1 (2.8%)	0 (0.0%)
>5	0 (0.0%)	0 (0.0%)	0 (0.0%)	0 (0.0%)	0 (0.0%)
**Household Chores**					
0	32 (88.9%)	31 (86.1%)	29 (80.6%)	27 (75.0%)	26 (72.2%)
1-2	3 (8.3%)	4 (11.1%)	6 (16.7%)	7 (19.4%)	7 (19.4%)
3-4	0 (0.0%)	0 (0.0%)	0 (0.0%)	1 (2.8%)	1 (2.8%)
>5	1 (2.8%)	1 (2.8%)	1 (2.8%)	1 (2.8%)	2 (5.6%)
**Personal support**					
0	33 (91.7%)	33 (91.7%)	33 (91.7%)	32 (88.9%)	31 (86.1%)
1-2	1 (2.8%)	1 (2.8%)	1 (2.8%)	1 (2.8%)	2 (5.6%)
3-4	1 (2.8%)	1 (2.8%)	1 (2.8%)	2 (5.6%)	1 (2.8%)
>5	1 (2.8%)	1 (2.8%)	1 (2.8%)	1 (2.8%)	2 (5.6%)
**Meals/ Grocery Shopping**					
0	29 (80.6%)	29 (80.6%)	29 (80.6%)	28 (77.8%)	26 (72.2%)
1-2	6 (16.7%)	5 (13.9%)	4 (11.1%)	6 (16.7%)	6 (16.7%)
3-4	0 (0.0%)	0 (0.0%)	1 (2.8%)	0 (0.0%)	2 (5.6%)
>5	1 (2.8%)	2 (5.6%)	2 (5.6%)	2 (5.6%)	2 (5.6%)
**Transport**					
0	33 (91.7%)	31 (86.1%)	34 (94.4%)	31 (86.1%)	31 (86.1%)
1-2	2 (5.6%)	4 (11.1%)	1 (2.8%)	4 (11.1%)	3 (8.3%)

3-4	1 (2.8%)	1 (2.8%)	1 (2.8%)	1 (2.8%)	2 (5.6%)
>5	0 (0.0%)	0 (0.0%)	0 (0.0%)	0 (0.0%)	0 (0.0%)
**Other**					
0					
1-2					
3-4					
>5					
