# Supplementary material for: Impact of Life Stressors on Myalgic Encephalomyelitis/Chronic Fatigue Syndrome Symptoms: An Australian Longitudinal Study
Source: Int J Environ Res Public Health. 2021 Oct 11;18(20):10614. doi: 10.3390/ijerph182010614 (PMC8535742; doi:10.3390/ijerph182010614)
Supplement: Supplementary file 1 [file ijerph-18-10614-s001.zip › Table S5. Frequency of access to volunteer services.pdf]

Table S5. Frequency of access to volunteer services

					N=36 (%)
	**0**	**1**	**2**	**3**	**4**
**Childminding/ Day-care**					
0	32 (88.9%)	31 (86.1%)	32 (88.9%)	33 (91.7%)	31 (86.1%)
1-2	2 (5.6%)	2 (5.6%)	2 (5.6%)	1 (2.8%)	3 (8.3%)
3-4	1 (2.8%)	1 (2.8%)	1 (2.8%)	2 (5.6%)	2 (5.6%)
>5	1 (2.8%)	1 (2.8%)	1 (2.8%)	0 (0.0%)	0 (0.0%)
**Household Chores**					
0	8 (22.2%)	8 (22.2%)	8 (22.2%)	9 (25.0%)	7 (19.4%)
1-2	10 (27.8%)	10 (27.8%)	8 (22.2%)	12 (33.3%)	12 (33.3%)
3-4	6 (16.7%)	8 (22.2%)	10 (27.8%)	5 (13.9%)	7 (19.4%)
>5	12 (33.3%)	10 (27.8%)	10 (27.8%)	10 (27.8%)	10 (27.8%)
**Personal support**					
0	19 (52.8%)	15 (41.7%)	17 (47.2%)	18 (50.0%)	19 (52.8%)
1-2	10 (27.8%)	11 (30.6%)	9 (25.0%)	8 (22.2%)	8 (22.2%)
3-4	2 (5.6%)	4 (11.1%)	5 (13.9%)	6 (16.7%)	6 (16.7%)
>5	5 (13.9%)	6 (16.7%)	5 (13.9%)	4 (11.1%)	3 (8.3%)
**Meals/ Grocery Shopping**					
0	12 (33.3%)	10 (27.8%)	10 (27.8%)	9 (25.0%)	10 (27.8%)
1-2	6 (16.7%)	9 (25.0%)	10 (27.8%)	12 (33.3%)	10 (27.8%)
3-4	6 (16.7%)	8 (22.2%)	6 (16.7%)	9 (25.0%)	6 (16.7%)
>5	12 (33.3%)	9 (25.0%)	10 (27.8%)	6 (16.7%)	10 (27.8%)
**Transport**					
0	18 (50.0%)	17 (47.2%)	16 (44.4%)	17 (47.2%)	18 (50.0%)
1-2	8 (22.2%)	11 (30.6%)	12 (33.3%)	9 (25.0%)	10 (27.8%)

3-4	4 (11.1%)	4 (11.1%)	5 (13.9%)	4 (11.1%)	4 (11.1%)
>5	6 (16.7%)	4 (11.1%)	3 (8.3%)	6 (16.7%)	4 (11.1%)
**Other**					
0					
1-2					
3-4					
>5					
