# Supplementary material for: Impact of Life Stressors on Myalgic Encephalomyelitis/Chronic Fatigue Syndrome Symptoms: An Australian Longitudinal Study
Source: Int J Environ Res Public Health. 2021 Oct 11;18(20):10614. doi: 10.3390/ijerph182010614 (PMC8535742; doi:10.3390/ijerph182010614)
Supplement: Supplementary file 1 [file ijerph-18-10614-s001.zip › Table S6. Frequency of symptoms.pdf]

Table S6. Frequency of symptoms

					N=36 (%)
	**0**	**1**	**2**	**3**	**4**
**Impaired thought, concentration, or difficulty processing information**					
None	0 (0.0%)	0 (0.0%)	2 (5.6%)	1 (2.8%)	0 (0.0%)
Mild	9 (25.0%)	11 (30.6%)	9 (25.0%)	8 (22.2%)	12 (33.3%)
Moderate	14 (38.9%)	11 (30.6%)	12 (33.3%)	16 (44.4%)	9 (25.0%)
Severe	11 (30.6%)	13 (36.1%)	11 (30.6%)	10 (27.8%)	14 (38.9%)
Extreme	2 (5.6%)	1 (2.8%)	2 (5.6%)	1 (2.8%)	1 (2.8%)
**Short or long-term memory loss**					
None	4 (11.1%)	4 (11.1%)	4 (11.1%)	3 (8.3%)	2 (5.6%)
Mild	14 (38.9%)	13 (36.1%)	15 (41.7%)	14 (38.9%)	13 (36.1%)
Moderate	12 (33.3%)	15 (41.7%)	11 (30.6%)	15 (41.7%)	13 (36.1%)
Severe	4 (11.1%)	4 (11.1%)	5 (13.9%)	4 (11.1%)	8 (22.2%)
Extreme	2 (5.6%)	0 (0.0%)	1 (2.8%)	0 (0.0%)	0 (0.0%)
**Headaches**					
None	9 (25.0%)	7 (19.4%)	5 (13.9%)	5 (13.9%)	3 (8.3%)
Mild	9 (25.0%)	12 (33.3%)	15 (41.7%)	13 (36.1%)	13 (36.1%)
Moderate	10 (27.8%)	12 (33.3%)	8 (22.2%)	9 (25.0%)	12 (33.3%)
Severe	7 (19.4%)	4 (11.1%)	7 (19.4%)	8 (22.2%)	7 (19.4%)
Extreme	1 (2.8%)	1 (2.8%)	1 (2.8%)	1 (2.8%)	1 (2.8%)
**Muscle pain**					
None	0 (0.0%)	0 (0.0%)	1 (2.8%)	1 (2.8%)	1 (2.8%)
Mild	8 (22.2%)	7 (19.4%)	9 (25.0%)	6 (16.7%)	6 (16.7%)
Moderate	19 (52.8%)	20 (55.6%)	14 (38.9%)	19 (52.8%)	15 (41.7%)

Severe	7 (19.4%)	8 (22.2%)	12 (33.3%)	9 (25.0%)	10 (27.8%)
Extreme	2 (5.6%)	1 (2.8%)	0 (0.0%)	1 (2.8%)	4 (11.1%)
**Joint pain**					
None	8 (22.2%)	7 (19.4%)	9 (25.0%)	8 (22.2%)	5 (13.9%)
Mild	10 (27.8%)	10 (27.8%)	6 (16.7%)	11 (30.6%)	11 (30.6%)
Moderate	14 (38.9%)	15 (41.7%)	15 (41.7%)	9 (25.0%)	11 (30.6%)
Severe	4 (11.1%)	4 (11.1%)	5 (13.9%)	8 (22.2%)	8 (22.2%)
Extreme	0 (0.0%)	0 (0.0%)	1 (2.8%)	0 (0.0%)	1 (2.8%)
**Sleep disturbances**					
None	0 (0.0%)	1 (2.8%)	1 (2.8%)	1 (2.8%)	2 (2.8%)
Mild	7 (19.4%)	4 (11.1%)	6 (16.7%)	8 (22.2%)	9 (25.0%)
Moderate	12 (33.3%)	15 (41.7%)	13 (36.1%)	11 (30.6%)	9 (25.0%)
Severe	15 (41.7%)	10 (27.8%)	11 (30.6%)	11 (30.6%)	12 (33.3%)
Extreme	2 (5.6%)	6 (16.7%)	5 (13.9%)	5 (13.9%)	5 (13.9%)
**Muscle weakness**					
None	2 (5.6%)	1 (2.8%)	2 (5.6%)	2 (5.6%)	1 (2.8%)
Mild	6 (16.7%)	12 (33.3%)	10 (27.8%)	10 (27.8%)	7 (19.4%)
Moderate	18 (50.0%)	13 (36.1%)	14 (38.9%)	12 (33.3%)	16 (44.4%)
Severe	8 (22.2%)	8 (22.2%)	8 (22.2%)	9 (25.0%)	8 (22.2%)
Extreme	2 (5.6%)	2 (5.6%)	2 (5.6%)	3 (8.3%)	4 (11.1%)
**Poor coordination**					
None	7 (19.4%)	4 (11.1%)	6 (16.7%)	6 (16.7%)	6 (16.7%)
Mild	14 (38.9%)	15 (41.7%)	14 (38.9%)	13 (36.1%)	10 (27.8%)
Moderate	12 (33.3%)	12 (33.3%)	11 (30.6%)	11 (30.6%)	16 (44.4%)
Severe	3 (8.3%)	5 (13.9%)	5 (13.9%)	6 (16.7%)	4 (11.1%)
Extreme	0 (0.0%)	0 (0.0%)	0 (0.0%)	0 (0.0%)	0 (0.0%)
**Sore throat**					

None	11 (30.6%)	11 (30.6%)	10 (27.8%)	11 (30.6%)	10 (27.8%)
Mild	11 (30.6%)	16 (44.4%)	13 (36.1%)	15 (41.7%)	16 (44.4%)
Moderate	12 (33.3%)	6 (16.7%)	9 (25.0%)	6 (16.7%)	5 (13.9%)
Severe	1 (2.8%)	2 (5.6%)	4 (11.1%)	4 (11.1%)	4 (11.1%)
Extreme	1 (2.8%)	1 (2.8%)	0 (0.0%)	0 (0.0%)	1 (2.8%)
**Tender lymph nodes**					
None	14 (38.9%)	16 (44.4%)	13 (36.1%)	17 (47.2%)	14 (38.9%)
Mild	12 (33.3%)	13 (36.1%)	10 (27.8%)	8 (22.2%)	11 (30.6%)
Moderate	9 (25.0%)	5 (13.9%)	11 (30.6%)	7 (19.4%)	7 (19.4%)
Severe	1 (2.8%)	2 (5.6%)	2 (5.6%)	4 (11.1%)	4 (11.1%)
Extreme	0 (0.0%)	0 (0.0%)	0 (0.0%)	0 (0.0%)	0 (0.0%)
**Nausea**					
None	14 (38.9%)	13 (36.1%)	17 (47.2%)	17 (47.2%)	13 (36.1%)
Mild	11 (30.6%)	14 (38.9%)	9 (25.0%)	7 (19.4%)	14 (38.9%)
Moderate	8 (22.2%)	7 (19.4%)	6 (16.7%)	10 (27.8%)	6 (16.7%)
Severe	3 (8.3%)	1 (2.8%)	4 (11.1%)	1 (2.8%)	1 (2.8%)
Extreme	0 (0.0%)	1 (2.8%)	0 (0.0%)	1 (2.8%)	2 (5.6%)
**Abdominal pain**					
None	12 (33.3%)	11 (30.6%)	13 (36.1%)	14 (38.9%)	9 (25.0%)
Mild	17 (47.2%)	17 (47.2%)	13 (36.1%)	11 (30.6%)	18 (50.0%)
Moderate	6 (16.7%)	7 (19.4%)	7 (19.4%)	9 (25.0%)	6 (16.7%)
Severe	1 (2.8%)	1 (2.8%)	3 (8.3%)	2 (5.6%)	3 (8.3%)
Extreme	0 (0.0%)	0 (0.0%)	0 (0.0%)	0 (0.0%)	0 (0.0%)
**Changes in frequency and volume of urination**					
None	13 (36.1%)	17 (47.2%)	16 (44.4%)	16 (44.4%)	16 (44.4%)
Mild	10 (27.8%)	10 (27.8%)	11 (30.6%)	11 (30.6%)	10 (27.8%)

Moderate	10 (27.8%)	5 (13.9%)	6 (16.7%)	5 (13.9%)	4 (11.1%)
Severe	2 (5.6%)	3 (8.3%)	2 (5.6%)	4 (11.1%)	5 (13.9%)
Extreme	1 (2.8%)	1 (2.8%)	1 (2.8%)	0 (0.0%)	1 (2.8%)
**Orthostatic intolerance (including POTS)**					
None	12 (33.3%)	10 (27.8%)	8 (22.2%)	10 (27.8%)	10 (27.8%)
Mild	12 (33.3%)	11 (30.6%)	11 (30.6%)	7 (19.4%)	7 (19.4%)
Moderate	5 (13.9%)	10 (27.8%)	9 (25.0%)	12 (33.3%)	9 (25.0%)
Severe	7 (19.4%)	4 (11.1%)	8 (22.2%)	6 (16.7%)	9 (25.0%)
Extreme	0 (0.0%)	1 (2.8%)	0 (0.0%)	1 (2.8%)	1 (2.8%)
**Intolerance to extreme temperatures**					
None	4 (11.1%)	6 (16.7%)	4 (11.1%)	8 (22.2%)	8 (22.2%)
Mild	11 (30.6%)	9 (25.0%)	13 (36.1%)	12 (33.3%)	10 (27.8%)
Moderate	12 (33.3%)	12 (33.3%)	12 (33.3%)	11 (30.6%)	11 (30.6%)
Severe	6 (16.7%)	6 (16.7%)	4 (11.1%)	3 (8.3%)	4 (11.1%)
Extreme	3 (8.3%)	3 (8.3%)	3 (8.3%)	2 (5.6%)	3 (8.3%)
**Other**					
None					
Mild					
Moderate					
Severe					
Extreme					
