# Supplementary material for: Impact of Life Stressors on Myalgic Encephalomyelitis/Chronic Fatigue Syndrome Symptoms: An Australian Longitudinal Study
Source: Int J Environ Res Public Health. 2021 Oct 11;18(20):10614. doi: 10.3390/ijerph182010614 (PMC8535742; doi:10.3390/ijerph182010614)
Supplement: Supplementary file 1 [file ijerph-18-10614-s001.zip › Table S7. Comparisons of Parameters using Friedman Test.pdf]

Table S7. Comparisons of Parameters using Friedman Test

	CATEGORY	SUB-CATEGORY	Month	N	Mean	Std. Deviation	Test Statistics		
							Chi-Square	df	Significance
FREQUENCY OF FAMILY, FINANCIAL AND WORK-RELATED PARAMETERS	WEEKLY WORK HOURS	N/A	0	36	0.611	0.494	6.400	4	0.171
			1		0.583	0.500			
			2		0.639	0.487			
			3		0.583	0.500			
			4		0.583	0.500			
		1-15	0	36	0.139	0.351	24.24	4	**0.000**
			1		0.167	0.378			
			2		0.361	0.487			
			3		0.194	0.401			
			4		0.167	0.378			
		16-24	0	36	0.194	0.401	31.52	4	**0.000**
			1		0.250	0.439			
			2		0.000	0.000			
			3		0.000	0.000			
			4		0.000	0.000			
		25-34	0	36	0.556	0.232	8.000	4	0.092
			1		0.000	0.000			
			2		0.000	0.000			
			3		0.000	0.000			
			4		0.000	0.000			
		≥ 35	0	36	0.000	0.000	.	4	.
			1		0.000	0.000			
			2		0.000	0.000			
			3		0.000	0.000			
			4		0.000	0.000			
		Yes	0	36	0.250	0.439	17.09	4	**0.002**

	**CHANGE IN WEEKLY WORK HOURS**		1		0.111	0.319			
			2		0.111	0.319			
			3		0.139	0.351			
			4		0.111	0.319			
		**No**	0	36	0.750	0.439	17.09	4	**0.002**
			1		0.889	0.319			
			2		0.889	0.319			
			3		0.861	0.351			
			4		0.889	0.319			
	**SUSTAINABLE INCOME**	**Yes**	0	36	0.528	0.506	8.571	4	0.073
			1		0.583	0.500			
			2		0.611	0.494			
			3		0.611	0.494			
			4		0.583	0.500			
		**No**	0	36	0.472	0.506	8.571	4	0.073
			1		0.417	0.500			
			2		0.389	0.494			
			3		0.389	0.494			
			4		0.417	0.500			
	**CHANGE IN HOUSEHOLD INCOME**	**Yes**	0	36	0.278	0.454	12.67	4	**0.013**
			1		0.167	0.378			
			2		0.222	0.422			
			3		0.222	0.422			
			4		0.139	0.351			
		**No**	0	36	0.722	0.454	12.67	4	**0.013**
			1		0.833	0.378			
			2		0.778	0.422			
			3		0.778	0.422			
			4		0.861	0.351			
		**Married**	0	36	0.444	0.504	.	4	.
			1		0.444	0.504			

	**LIVING ARRANGMENT**		2		0.444	0.504			
			3		0.444	0.504			
			4		0.444	0.504			
		**Single**	0	36	0.250	0.439	.	4	**.**
			1		0.250	0.439			
			2		0.250	0.439			
			3		0.250	0.439			
			4		0.250	0.439			
		**Divorced/Separated**	0	36	0.056	0.232	.	4	.
			1		0.056	0.232			
			2		0.056	0.232			
			3		0.056	0.232			
			4		0.056	0.232			
		**Widow/er**	0	36	0.000	0.000	.	4	.
			1		0.000	0.000			
			2		0.000	0.000			
			3		0.000	0.000			
			4		0.000	0.000			
		**Single with Children**	0	36	0.028	0.167	.	4	.
			1		0.028	0.167			
			2		0.028	0.167			
			3		0.028	0.167			
			4		0.028	0.167			
		**Married/De Facto with Children**	0	36	0.222	0.422	.	4	.
			1		0.222	0.422			
			2		0.222	0.422			
			3		0.222	0.422			
			4		0.222	0.422			
		**0**	0	36	0.583	0.500	15.20	4	**0.004**
			1		0.500	0.507			

			2		0.667	0.478			
			3		0.528	0.506			
			4		0.611	0.494			
		**1-2**	0	36	0.417	0.500	15.14	4	**0.004**
			1		0.500	0.507			
			2		0.333	0.478			
			3		0.417	0.500			
			4		0.361	0.487			
	**GP**	**3-4**	0	36	0.000	0.000	6.400	4	0.171
			1		0.000	0.000			
			2		0.000	0.000			
			3		0.056	0.232			
			4		0.028	0.167			
		**>5**	0	36	0.000	0.000	.	4	.
**ACCESS TO HEALTHCARE SERVICES**			1		0.000	0.000			
			2		0.000	0.000			
			3		0.000	0.000			
			4		0.000	0.000			
	**NURSE**	**0**	0	36	0.972	0.167	8.500	4	0.075
			1		0.889	0.319			
			2		0.889	0.319			
			3		0.944	0.232			
			4		0.917	0.280			
		**1-2**	0	36	0.111	0.319	13.33	4	**0.010**
			1		0.222	0.422			
			2		0.167	0.378			
			3		0.111	0.317			
			4		0.083	0.280			
		**3-4**	0	36	0.000	0.000	4.000	4	0.406
			1		0.000	0.000			
			2		0.028	0.167			

			3		0.000	0.000			
			4		0.000	0.000			
		**>5**	0	36	0.000	0.000	.	4	.
			1		0.000	0.000			
			2		0.000	0.000			
			3		0.000	0.000			
			4		0.000	0.000			
	**PATHOLOGIST**	**0**	0	36	0.889	0.319	12.00	4	**0.017**
			1		0.778	0.422			
			2		0.806	0.401			
			3		0.861	0.351			
			4		0.778	0.422			
		**1-2**	0	36	0.111	0.319	13.33	4	**0.010**
			1		0.222	0.422			
			2		0.167	0.378			
			3		0.111	0.319			
			4		0.083	0.280			
		**3-4**	0	36	0.000	0.000	4.000	4	0.406
			1		0.000	0.000			
			2		0.000	0.000			
			3		0.028	0.167			
			4		0.028	0.167			
		**>5**	0	36	0.000	0.000	4.000	4	0.406
			1		0.000	0.000			
			2		0.028	0.167			
			3		0.000	0.000			
			4		0.000	0.000			
		**0**	0	36	0.833	0.378	8.500	4	0.075
			1		0.778	0.422			
			2		0.806	0.401			
			3		0.861	0.351			

			4		0.778	0.422			
	MEDICAL SPECIALIST	**1-2**	0	36	0.111	0.319	10.22	4	**0.037**
			1		0.194	0.401			
			2		0.167	0.378			
			3		0.111	0.319			
			4		0.194	0.401			
		**3-4**	0	36	0.056	0.232	5.000	4	0.287
			1		0.028	0.167			
			2		0.000	0.000			
			3		0.028	0.167			
			4		0.028	0.167			
		**>5**	0	36	0.000	0.000	4.000	4	0.406
			1		0.000	0.000			
			2		0.028	0.167			
			3		0.000	0.000			
			4		0.000	0.000			
	OCCUPATIONAL THERAPIST	**0**	0	36	0.917	0.280	11.20	4	**0.024**
			1		0.972	0.167			
			2		0.972	0.167			
			3		0.917	0.280			
			4		0.861	0.351			
		**1-2**	0	36	0.083	0.280	11.20	4	**0.024**
			1		0.028	0.167			
			2		0.028	0.167			
			3		0.083	0.280			
			4		0.139	0.351			
		**3-4**	0	36	0.000	0.000	.	4	.
			1		0.000	0.000			
			2		0.000	0.000			
			3		0.000	0.000			
			4		0.000	0.000			

		**>5**	0	36	0.000	0.000	.	4	.
			1		0.000	0.000			
			2		0.000	0.000			
			3		0.000	0.000			
			4		0.000	0.000			
	**PHYSIOTHERAPIST**	**0**	0	36	0.917	0.280	14.33	4	**0.006**
			1		0.889	0.319			
			2		0.917	0.280			
			3		0.778	0.422			
			4		0.861	0.351			
		**1-2**	0	36	0.083	0.280	14.33	4	**0.006**
			1		0.111	0.319			
			2		0.083	0.280			
			3		0.222	0.422			
			4		0.139	0.351			
		**3-4**	0	36	0.000	0.000	.	4	.
			1		0.000	0.000			
			2		0.000	0.000			
			3		0.000	0.000			
			4		0.000	0.000			
		**>5**	0	36	0.000	0.000	.	4	.
			1		0.000	0.000			
			2		0.000	0.000			
			3		0.000	0.000			
			4		0.000	0.000			
		**0**	0	36	0.711	0.454	16.53	4	**0.002**
			1		0.639	0.487			
			2		0.694	0.467			
			3		0.806	0.401			
			4		0.639	0.487			
		**1-2**	0	36	0.278	0.454	16.53	4	**0.002**

	OTHER		1		0.361	0.487			
			2		0.306	0.467			
			3		0.194	0.401			
			4		0.361	0.487			
		**3-4**	0	36	0.000	0.000	.	4	.
			1		0.000	0.000			
			2		0.000	0.000			
			3		0.000	0.000			
			4		0.000	0.000			
		**>5**	0	36	0.000	0.000	.	4	.
			1		0.000	0.000			
			2		0.000	0.000			
			3		0.000	0.000			
			4		0.000	0.000			
	**IMPAIRED THOUGHT, CONCENTRATION, OR DIFFICULTY PROCESSING INFORMATION**	**NONE**	0	36	0.000	0.000	6.400	4	0.171
			1		0.000	0.000			
			2		0.056	0.232			
			3		0.028	0.167			
			4		0.000	0.000			
		**MILD**	0	36	0.250	0.439	21.25	4	**0.000**
			1		0.278	0.454			
			2		0.306	0.467			
			3		0.444	0.504			
			4		0.250	0.439			
		**MODERATE**	0	36	0.389	0.494	17.18	4	**0.002**
			1		0.306	0.467			
			2		0.333	0.478			
			3		0.444	0.504			
			4		0.250	0.439			
		**SEVERE**	0	36	0.306	0.467	10.80	4	**0.029**

			1		0.361	0.487			
			2		0.306	0.487			
			3		0.278	0.454			
			4		0.389	0.494			
		**EXTREME**	0	36	0.056	0.232	4.000	4	0.406
			1		0.028	0.167			
			2		0.056	0.232			
			3		0.028	0.167			
			4		0.028	0.167			
	**SHORT OR LONG-TERM MEMORY LOSS**	**NONE**	0	36	0.111	0.319	6.400	4	0.171
			1		0.111	0.319			
			2		0.111	0.319			
			3		0.083	0.280			
			4		0.056	0.232			
		**MILD**	0	36	0.389	0.494	5.600	4	0.231
			1		0.361	0.487			
			2		0.417	0.500			
			3		0.389	0.494			
			4		0.361	0.487			
		**MODERATE**	0	36	0.333	0.478	11.64	4	**0.020**
			1		0.417	0.500			
			2		0.306	0.467			
			3		0.417	0.500			
			4		0.361	0.487			
		**SEVERE**	0	36	0.111	0.319	13.33	4	**0.010**
			1		0.111	0.319			
			2		0.139	0.351			
			3		0.111	0.319			
			4		0.222	0.422			
		**EXTREME**	0	36	0.056	0.232	6.400	4	0.171
			1		0.000	0.000			

			2		0.028	0.167			
			3		0.000	0.000			
			4		0.000	0.000			
			1		0.000	0.000			
			2		0.000	0.000			
			3		0.028	0.167			
			4		0.028	0.167			
	HEADACHES	**NONE**	0	36	0.139	0.351	5.600	4	0.231
			1		0.111	0.319			
			2		0.111	0.319			
			3		0.139	0.351			
			4		0.083	0.280			
		**MILD**	0	36	0.250	0.439	14.86	4	**0.005**
			1		0.194	0.401			
			2		0.139	0.351			
			3		0.139	0.351			
			4		0.083	0.280			
		**MODERATE**	0	36	0.278	0.454	11.636	4	**0.020**
			1		0.333	0.478			
			2		0.222	0.422			
			3		0.250	0.439			
			4		0.333	0.478			
		**SEVERE**	0	36	0.194	0.401	11.50	4	**0.021**
			1		0.111	0.319			
			2		0.194	0.401			
			3		0.222	0.422			
			4		0.194	0.401			
		**EXTREME**	0	36	0.028	0.167	.	4	.
			1		0.028	0.167			
			2		0.028	0.167			
			3		0.028	0.167			

			4		0.028	0.167			
	MUSCLE PAIN	**NONE**	0	36	0.000	0.000	4.000	4	0.406
			1		0.000	0.000			
			2		0.028	0.167			
			3		0.028	0.167			
			4		0.028	0.167			
		**MILD**	0	36	0.222	0.422	8.500	4	0.075
			1		0.194	0.401			
			2		0.250	0.439			
			3		0.167	0.378			
			4		0.167	0.78			
		**MODERATE**	0	36	0.528	0.506	18.25	4	**0.001**
			1		0.556	0.504			
			2		0.389	0.494			
			3		0.528	0.506			
			4		0.417	0.500			
		**SEVERE**	0	36	0.194	0.401	12.33	4	**0.015**
			1		0.222	0.422			
			2		0.333	0.478			
			3		0.250	0.439			
			4		0.278	0.454			
		**EXTREME**	0	36	0.056	0.232	10.22	4	**0.037**
			1		0.028	0.167			
			2		0.000	0.000			
			3		0.028	0.167			
			4		0.111	0.319			
		**NONE**	0	36	0.222	0.422	10.22	4	**0.037**
			1		0.194	0.401			
			2		0.250	0.439			
			3		0.222	0.422			
			4		0.139	0.351			

	JOINT PAIN	**MILD**	0	36	0.278	0.454	15.64	4	**0.004**
			1		0.278	0.454			
			2		0.167	0.378			
			3		0.306	0.378			
			4		0.306	0.467			
		**MODERATE**	0	36	0.389	0.494	18.00	4	**0.001**
			1		0.417	0.500			
			2		0.417	0.500			
			3		0.250	0.439			
			4		0.306	0.467			
		**SEVERE**	0	36	0.389	0.494	18.00	4	**0.001**
			1		0.417	0.500			
			2		0.417	0.500			
			3		0.250	0.439			
			4		0.306	0.467			
		**EXTREME**	0	36	0.000	0.000	4.000	4	0.406
			1		0.000	0.000			
			2		0.028	0.167			
			3		0.000	0.000			
			4		0.028	0.167			
	SLEEP DISTURBANCES	**NONE**	0	36	0.000	0.000	5.000	4	0.287
			1		0.028	0.167			
			2		0.028	0.167			
			3		0.028	0.167			
			4		0.028	0.167			
		**MILD**	0	36	0.194	0.401	12.33	4	**0.015**
			1		0.111	0.319			
			2		0.167	0.378			
			3		0.222	0.422			
			4		0.250	0.439			
		**MODERATE**	0	36	0.333	0.478	14.29	4	**0.006**

			1		0.417	0.500			
			2		0.361	0.487			
			3		0.306	0.467			
			4		0.250	0.439			
		**SEVERE**	0	36	0.111	0.319	14.00	4	**0.007**
			1		0.111	0.319			
			2		0.139	0.351			
			3		0.222	0.422			
			4		0.222	0.422			
		**EXTREME**	0	36	0.000	0.000	4.000	4	0.406
			1		0.000	0.000			
			2		0.028	0.167			
			3		0.000	0.000			
			4		0.028	0.167			
			1		0.000	0.000			
			2		0.000	0.000			
			3		0.000	0.000			
			4		0.000	0.000			
	**SORE THROAT**	**NONE**	0	36	0.306	0.467	4.000	4	0.406
			1		0.306	0.467			
			2		0.278	0.454			
			3		0.306	0.467			
			4		0.278	0.454			
		**MILD**	0	36	0.306	0.467	14.46	4	**0.006**
			1		0.444	0.504			
			2		0.361	0.487			
			3		0.417	0.500			
			4		0.444	0.504			
		**MODERATE**	0	36	0.333	0.478	19.53	4	**0.001**
			1		0.167	0.378			
			2		0.250	0.439			

			3		0.167	0.378			
			4		0.139	0.351			
		**SEVERE**	0	36	0.028	0.167	10.00		**0.040**
			1		0.056	0.232			
			2		0.111	0.319			
			3		0.111	0.319			
			4		0.111	0.319			
		**EXTREME**	0	36	0.028	0.167	4.000	4	0.406
			1		0.028	0.167			
			2		0.000	0.000			
			3		0.000	0.000			
			4		0.028	0.167			
	**TENDER LYMPH NODES**	**NONE**	0	36	0.389	0.494	10.80	4	**0.029**
			1		0.444	0.504			
			2		0.361	0.487			
			3		0.472	0.506			
			4		0.389	0.494			
		**MILD**	0	36	0.333	0.478	12.33	4	**0.015**
			1		0.361	0.487			
			2		0.278	0.454			
			3		0.222	0.422			
			4		0.306	0.467			
		**MODERATE**	0	36	0.250	0.439	14.86	4	**0.005**
			1		0.139	0.351			
			2		0.306	0.467			
			3		0.194	0.401			
			4		0.194	0.401			
		**SEVERE**	0	36	0.028	0.167	9.000		0.061
			1		0.556	0.232			
			2		0.556	0.232			
			3		0.111	0.319			

			4		0.111	0.319			
		**EXTREME**	0	36	0.000	0.000	.	4	.
			1		0.000	0.000			
			2		0.000	0.000			
			3		0.000	0.000			
			4		0.000	0.000			
	**NAUSEA**	**NONE**	0	36	0.389	0.494	14.00	4	**0.007**
			1		0.361	0.487			
			2		0.472	0.506			
			3		0.472	0.506			
			4		0.361	0.487			
		**MILD**	0	36	0.306	0.467	20.00	4	**0.000**
			1		0.389	0.494			
			2		0.250	0.439			
			3		0.194	0.401			
			4		0.389	0.494			
		**MODERATE**	0	36	0.222	0.422	11.20	4	**0.024**
			1		0.194	0.401			
			2		0.167	0.378			
			3		0.278	0.454			
			4		0.167	0..378			
		**SEVERE**	0	36	0.083	0.280	10.00	4	**0.040**
			1		0.028	0.167			
			2		0.111	0.319			
			3		0.028	0.167			
			4		0.028	0.167			
		**EXTREME**	0	36	0.000	0.000	5.600	4	0.231
			1		0.028	0.167			
			2		0.000	0.000			
			3		0.028	0.167			
			4		0.056	0.232			

	ABDOMINAL PAIN	**NONE**	0	36	0.333	0.478	12.33	4	**0.015**
			1		0.306	0.467			
			2		0.361	0.487			
			3		0.389	0.494			
			4		0.250	0.439			
		**MILD**	0	36	0.472	0.506	20.44	4	**0.000**
			1		0.472	0.506			
			2		0.361	0.487			
			3		0.306	0.467			
			4		0.500	0.507			
		**MODERATE**	0	36	0.167	0.378	8.571	4	0.073
			1		0.194	0.401			
			2		0.194	0.401			
			3		0.250	0.439			
			4		0.167	0.378			
		**SEVERE**	0	36	0.028	0.167	6.667	4	0.155
			1		0.028	0.167			
			2		0.083	0.280			
			3		0.056	0.232			
			4		0.083	0.280			
		**EXTREME**	0	36	0.000	0.000	.	4	.
			1		0.000	0.000			
			2		0.000	0.000			
			3		0.000	0.000			
			4		0.000	0.000			
		**NONE**	0	36	0.361	0.487	11.50	4	**0.021**
			1		0.472	0.506			
			2		0.444	0.504			
			3		0.444	0.504			
			4		0.444	0.504			
		**MILD**	0	36	0.278	0.454	4.000	4	0.406

			1		0.278	0.454			
			2		0.306	0.467			
			3		0.306	0.467			
			4		0.278	0.454			
	**CHANGES IN FREQUENCY AND VOLUME OF URINATION**	**MODERATE**	0	36	0.278	0.454	16.92	4	**0.002**
			1		0.139	0.351			
			2		0.167	0.378			
			3		0.139	0.351			
			4		0.111	0.319			
		**SEVERE**	0	36	0.056	0.232	8.500	4	0.075
			1		0.083	0.280			
			2		0.056	0.232			
			3		0.111	0.319			
			4		0.139	0.351			
		**EXTREME**	0	36	0.028	0.167	4.000	4	0.406
			1		0.028	0.167			
			2		0.028	0.167			
			3		0.000	0.000			
			4		0.028	0.167			
		**NONE**	0	36	0.333	0.478	10.00	4	**0.040**
			1		0.278	0.454			
			2		0.222	0.422			
			3		0.278	0.454			
			4		0.278	0.454			
		**MILD**	0	36	0.333	0.478	16.57	4	**0.002**
			1		0.351	0.467			
			2		0.351	0.467			
			3		0.194	0.401			
			4		0.194	0.401			
		**MODERATE**	0	36	0.139	0.351	17.33	4	**0.002**
			1		0.278	0.454			

	**ORTHOSTATIC INTOLERANCE (including POTS)**		2		0.250	0.439			
			3		0.333	0.478			
			4		0.250	0.439			
		**SEVERE**	0	36	0.194	0.401	12.33	4	**0.015**
			1		0.111	0.319			
			2		0.222	0.422			
			3		0.167	0.378			
			4		0.250	0.439			
		**EXTREME**	0	36	0.000	0.000	4.000	4	0.406
			1		0.028	0.167			
			2		0.000	0.000			
			3		0.028	0.167			
			4		0.028	0.167			
	**INTOLERANCE TO EXTREME TEMPERATURES**	**NONE**	0	36	0.111	0.319	18.22	4	**0.001**
			1		0.167	0.378			
			2		0.083	0.280			
			3		0.222	0.422			
			4		0.278	0.454			
		**MILD**	0	36	0.444	0.504	13.23	4	**0.010**
			1		0.333	0.478			
			2		0.417	0.500			
			3		0.361	0.487			
			4		0.306	0.467			
		**MODERATE**	0	36	0.278	0.454	12.33	4	**0.015**
			1		0.333	0.478			
			2		0.417	0.500			
			3		0.361	0.487			
			4		0.306	0.467			
		**SEVERE**	0	36	0.139	0.351	12.73	4	**0.013**
			1		0.111	0.319			
			2		0.028	0.167			

			3		0.111	0.319			
			4		0.028	0.167			
		**EXTREME**	0	36	0.056	0.232	4.000	4	0.406
			1		0.056	0.232			
			2		0.056	0.232			
			3		0.056	0.232			
			4		0.083	0.280			
			1		0.056	0.232			
			2		0.028	0.167			
			3		0.028	0.167			
			4		0.000	0.000			
