# Supplementary material for: Impact of Life Stressors on Myalgic Encephalomyelitis/Chronic Fatigue Syndrome Symptoms: An Australian Longitudinal Study
Source: Int J Environ Res Public Health. 2021 Oct 11;18(20):10614. doi: 10.3390/ijerph182010614 (PMC8535742; doi:10.3390/ijerph182010614)
Supplement: Supplementary file 1 [file ijerph-18-10614-s001.zip › Table S8. Comparisons Wilcoxon Post Hoc Test.pdf]

Table S8. Comparisons Wilcoxon Post Hoc Test

	VARIABLES	Months	Z statistic	P Value
WEEKLY WORK HOURS	1-15	Baseline vs Month 1	-1.000	0.317
		Baseline vs Month 2	-2.828	**0.005**
		Baseline vs Month 3	-1.414	0.157
		Baseline vs Month 4	-1.000	0.317
		Month 1 vs Month 2	-2.646	**0.008**
		Month 1 vs Month 3	-1.000	0.317
		Month 1 vs Month 4	0.000	1.000
		Month 2 vs Month 3	-2.449	0.014
		Month 2 vs Month 4	-2.646	**0.008**
		Month 3 vs Month 4	-1.000	0.317
	16-24	Baseline vs Month 1	-2.236	**0.025**
		Baseline vs Month 2	-2.236	**0.025**
		Baseline vs Month 3	-2.000	**0.046**
		Baseline vs Month 4	-2.236	**0.025**
		Month 1 vs Month 2	0.000	1.000
		Month 1 vs Month 3	-1.000	0.317
		Month 1 vs Month 4	0.000	1.000
		Month 2 vs Month 3	-1.000	0.317
		Month 2 vs Month 4	0.000	1.000
		Month 3 vs Month 4	-1.000	0.317
CHANGE IN WEEKLY WORK HOURS	YES	Baseline vs Month 1	-2.236	**0.025**
		Baseline vs Month 2	-2.236	**0.025**
		Baseline vs Month 3	-2.000	**0.046**
		Baseline vs Month 4	-2.236	**0.025**
		Month 1 vs Month 2	0.000	1.000
		Month 1 vs Month 3	-1.000	0.317

		Month 1 vs Month 4	0.000	1.000
		Month 2 vs Month 3	-1.000	0.317
		Month 2 vs Month 4	0.000	1.000
		Month 3 vs Month 4	-1.000	0.317
	**NO**	Baseline vs Month 1	-2.236	**0.025**
		Baseline vs Month 2	-2.236	**0.025**
		Baseline vs Month 3	-2.000	**0.046**
		Baseline vs Month 4	-2.236	**0.025**
		Month 1 vs Month 2	0.000	1.000
		Month 1 vs Month 3	-1.000	0.317
		Month 1 vs Month 4	0.000	1.000
		Month 2 vs Month 3	-1.000	0.317
		Month 2 vs Month 4	0.000	1.000
		Month 3 vs Month 4	-1.000	0.317
**CHANGE IN HOUSEHOLD INCOME**	**YES**	Baseline vs Month 1	-2.000	**0.046**
		Baseline vs Month 2	-1.414	0.157
		Baseline vs Month 3	-1.414	0.157
		Baseline vs Month 4	-2.236	0.025
		Month 1 vs Month 2	-1.414	0.157
		Month 1 vs Month 3	-1.414	0.157
		Month 1 vs Month 4	-1.000	0.317
		Month 2 vs Month 3	0.000	1.000
		Month 2 vs Month 4	-1.732	0.083
		Month 3 vs Month 4	-1.732	0.083
	**NO**	Baseline vs Month 1	-2.000	**0.046**
		Baseline vs Month 2	-1.414	0.157
		Baseline vs Month 3	-1.414	0.157
		Baseline vs Month 4	-2.236	**0.025**
		Month 1 and Month 2	-1.414	0.157
		Month 1 and Month 3	-1.414	0.157
		Month 1 and Month 4	-1.000	0.317

		Month 2 and Month 3	0.000	1.000
		Month 2 and Month 4	-1.732	0.083
		Month 3 and Month 4	-1.732	0.083
**GP**	**0**	Baseline vs Month 1	-1.732	0.083
		Baseline vs Month 2	-1.732	0.083
		Baseline vs Month 3	-1.414	0.157
		Baseline vs Month 4	-1.000	0.317
		Month 1 vs Month 2	-2.449	**0.014**
		Month 1 vs Month 3	-1.000	0.317
		Month 1 vs Month 4	-2.000	**0.046**
		Month 2 vs Month 3	-2.236	**0.025**
		Month 2 vs Month 4	-1.414	0.157
		Month 3 vs Month 4	-1.732	0.083
	**1-2**	Baseline vs Month 1	-1.732	0.083
		Baseline vs Month 2	-1.732	0.083
		Baseline vs Month 3	0.000	1.000
		Baseline vs Month 4	-1.414	0.157
		Month 1 and Month 2	-2.449	**0.014**
		Month 1 and Month 3	-1.732	0.083
		Month 1 and Month 4	-2.236	**0.025**
		Month 2 and Month 3	-1.732	0.083
		Month 2 and Month 4	-1.000	0.317
		Month 3 and Month 4	-1.414	0.157
**NURSE**	**1-2**	Baseline vs Month 1	-2.000	**0.046**
		Baseline vs Month 2	-1.414	0.157
		Baseline vs Month 3	0.000	1.000
		Baseline vs Month 4	-1.000	0.317
		Month 1 and Month 2	-1.414	0.157
		Month 1 and Month 3	-2.000	**0.046**
		Month 1 and Month 4	-2.236	**0.025**

		Month 2 and Month 3	-1.414	0.157
		Month 2 and Month 4	-1.732	0.083
		Month 3 and Month 4	-1.000	0.317
**PATHOLOGIST**	**0**	Baseline vs Month 1	-2.000	**0.046**
		Baseline vs Month 2	-1.732	0.083
		Baseline vs Month 3	-1.000	0.317
		Baseline vs Month 4	-2.000	**0.046**
		Month 1 and Month 2	-1.000	0.317
		Month 1 and Month 3	-1.732	**0.083**
		Month 1 and Month 4	0.000	1.000
		Month 2 and Month 3	-1.414	0.157
		Month 2 and Month 4	-1.000	0.317
		Month 3 and Month 4	-1.732	0.083
	**1-2**	Baseline vs Month 1	-2.000	**0.046**
		Baseline vs Month 2	-1.414	0.157
		Baseline vs Month 3	0.000	1.000
		Baseline vs Month 4	-1.000	0.317
		Month 1 and Month 2	-1.414	0.157
		Month 1 and Month 3	-2.000	**0.046**
		Month 1 and Month 4	-2.236	**0.025**
		Month 2 and Month 3	-1.414	0.157
		Month 2 and Month 4	-1.732	0.083
		Month 3 and Month 4	-1.000	0.317
**MEDICAL SPECIALIST**	**1-2**	Baseline vs Month 1	-1.732	0.083
		Baseline vs Month 2	-1.414	0.157
		Baseline vs Month 3	0.000	1.000
		Baseline vs Month 4	-1.732	0.083
		Month 1 and Month 2	-1.000	0.317
		Month 1 and Month 3	-1.732	0.083
		Month 1 and Month 4	0.000	1.000
		Month 2 and Month 3	-1.414	0.157

		Month 2 and Month 4	-1.000	0.317
		Month 3 and Month 4	-1.732	0.083
**OCCUPATIONAL THERAPIST**	**0**	Baseline vs Month 1	-1.414	0.157
		Baseline vs Month 2	-1.414	0.157
		Baseline vs Month 3	0.000	1.000
		Baseline vs Month 4	-1.414	0.157
		Month 1 and Month 2	0.000	1.000
		Month 1 and Month 3	-1.414	0.157
		Month 1 and Month 4	-2.000	**0.046**
		Month 2 and Month 3	-1.414	0.157
		Month 2 and Month 4	-2.000	**0.046**
		Month 3 and Month 4	-1.414	0.157
	**1-2**	Baseline vs Month 1	-1.414	0.157
		Baseline vs Month 2	-1.414	0.157
		Baseline vs Month 3	0.000	1.000
		Baseline vs Month 4	-1.414	0.157
		Month 1 and Month 2	0.000	1.000
		Month 1 and Month 3	-1.414	0.157
		Month 1 and Month 4	-2.000	**0.046**
		Month 2 and Month 3	-1.414	0.157
		Month 2 and Month 4	-2.000	**0.046**
		Month 3 and Month 4	-1.414	0.157
**PHYSIOTHERAPIST**	**0**	Baseline vs Month 1	-1.000	0.317
		Baseline vs Month 2	0.000	1.000
		Baseline vs Month 3	-2.236	**0.025**
		Baseline vs Month 4	-1.414	0.157
		Month 1 and Month 2	-1.000	0.317
		Month 1 and Month 3	-2.000	**0.046**
		Month 1 and Month 4	-1.000	0.317
		Month 2 and Month 3	-2.236	**0.025**
		Month 2 and Month 4	-1.414	0.157

		Month 3 and Month 4	-1.732	0.083
	**1-2**	Baseline vs Month 1	-1.000	0.317
		Baseline vs Month 2	0.000	1.000
		Baseline vs Month 3	-2.236	0.025
		Baseline vs Month 4	-1.414	0.157
		Month 1 and Month 2	-1.000	0.317
		Month 1 and Month 3	-2.000	**0.046**
		Month 1 and Month 4	-1.000	0.317
		Month 2 and Month 3	-2.236	**0.025**
		Month 2 and Month 4	-1.414	0.157
		Month 3 and Month 4	-1.732	0.083
**OTHER**	**0**	Baseline vs Month 1	-1.732	0.083
		Baseline vs Month 2	-1.000	0.317
		Baseline vs Month 3	-1.732	0.083
		Baseline vs Month 4	-1.732	0.083
		Month 1 and Month 2	-1.414	0.157
		Month 1 and Month 3	-2.449	**0.014**
		Month 1 and Month 4	0.000	1.000
		Month 2 and Month 3	-2.000	**0.046**
		Month 2 and Month 4	-1.414	0.157
		Month 3 and Month 4	-2.449	**0.014**
	**1-2**	Baseline vs Month 1	-1.732	0.083
		Baseline vs Month 2	-1.000	0.317
		Baseline vs Month 3	-1.732	0.083
		Baseline vs Month 4	-1.732	0.083
		Month 1 and Month 2	-1.414	0.157
		Month 1 and Month 3	-2.449	**0.014**
		Month 1 and Month 4	0.000	1.000
		Month 2 and Month 3	-2.000	**0.046**
		Month 2 and Month 4	-1.414	0.157
		Month 3 and Month 4	-2.449	0.014

**HOUSEHOLD CHORES (P)**	**1-2**	Baseline vs Month 1	-0.577	0.564
		Baseline vs Month 2	-1.342	0.180
		Baseline vs Month 3	-2.121	**0.034**
		Baseline vs Month 4	-2.264	**0.024**
		Month 1 and Month 2	-1.414	0.157
		Month 1 and Month 3	-1.667	0.096
		Month 1 and Month 4	-1.930	0.054
		Month 2 and Month 3	-1.134	0.257
		Month 2 and Month 4	-1.667	0.096
		Month 3 and Month 4	-1.732	0.83
**MEALS (P)**	**0**	Baseline vs Month 1	0.000	1.000
		Baseline vs Month 2	0.000	1.000
		Baseline vs Month 3	-1.000	0.317
		Baseline vs Month 4	-1.732	0.083
		Month 1 and Month 2	0.000	1.000
		Month 1 and Month 3	-1.000	0.317
		Month 1 and Month 4	-1.732	0.083
		Month 2 and Month 3	-1.000	0.317
		Month 2 and Month 4	-1.732	0.083
		Month 3 and Month 4	-1.414	0.157
**TRANSPORT (P)**	**0**	Baseline vs Month 1	-1.414	0.157
		Baseline vs Month 2	-1.000	0.317
		Baseline vs Month 3	-1.414	0.157
		Baseline vs Month 4	-1.414	0.157
		Month 1 and Month 2	-1.732	0.083
		Month 1 and Month 3	0.000	1.000
		Month 1 and Month 4	0.000	1.000
		Month 2 and Month 3	-1.732	0.083
		Month 2 and Month 4	-1.732	0.083
		Month 3 and Month 4	0.000	1.000

**HOUSEHOLD CHORES (UP)**	**1-2**	Baseline vs Month 1	0.000	1.000
		Baseline vs Month 2	-1.414	0.157
		Baseline vs Month 3	-1.414	0.157
		Baseline vs Month 4	-1.414	0.157
		Month 1 and Month 2	-1.414	0.157
		Month 1 and Month 3	-1.414	0.157
		Month 1 and Month 4	-1.414	0.157
		Month 2 and Month 3	-2.000	**0.046**
		Month 2 and Month 4	-2.000	**0.046**
		Month 3 and Month 4	0.000	1.000
	**3-4**	Baseline vs Month 1	-1.414	0.157
		Baseline vs Month 2	-2.000	**0.046**
		Baseline vs Month 3	-1.000	0.317
		Baseline vs Month 4	-1.000	0.317
		Month 1 and Month 2	-1.414	0.157
		Month 1 and Month 3	-1.732	0.083
		Month 1 and Month 4	-1.000	1.000
		Month 2 and Month 3	-2.236	**0.025**
		Month 2 and Month 4	-1.732	0.083
		Month 3 and Month 4	-1.414	0.157
**PERSONAL SUPPORT (UP)**	**0**	Baseline vs Month 1	-2.000	0.046
		Baseline vs Month 2	-1.414	0.157
		Baseline vs Month 3	-1.000	0.317
		Baseline vs Month 4	0.000	1.000
		Month 1 and Month 2	-1.414	0.157
		Month 1 and Month 3	-1.732	**0.046**
		Month 1 and Month 4	-2.000	**0.046**
		Month 2 and Month 3	-1.000	0.317
		Month 2 and Month 4	-1.414	0.157
		Month 3 and Month 4	-1.000	0.317
		Baseline vs Month 1	-1.414	0.157

	**3-4**	Baseline vs Month 2	-1.732	**0.046**
		Baseline vs Month 3	-2.000	**0.046**
		Baseline vs Month 4	-2.000	**0.046**
		Month 1 and Month 2	-1.000	0.317
		Month 1 and Month 3	-1.414	0.157
		Month 1 and Month 4	-1.414	0.157
		Month 2 and Month 3	-1.000	0.317
		Month 2 and Month 4	-1.000	0.317
		Month 3 and Month 4	0.000	1.000
	**>5**	Baseline vs Month 1	-1.414	0.157
		Baseline vs Month 2	0.000	1.000
		Baseline vs Month 3	-1.000	0.317
		Baseline vs Month 4	-1.414	0.157
		Month 1 and Month 2	-1.414	0.157
		Month 1 and Month 3	-1.732	0.083
		Month 1 and Month 4	-2.000	**0.046**
		Month 2 and Month 3	-1.000	0.317
		Month 2 and Month 4	-1.414	0.157
		Month 3 and Month 4	-1.000	0.317
**MEALS (UP)**	**1-2**	Baseline vs Month 1	-1.732	0.086
		Baseline vs Month 2	-2.000	**0.046**
		Baseline vs Month 3	-2.000	**0.046**
		Baseline vs Month 4	-1.000	0.317
		Month 1 and Month 2	-1.732	0.083
		Month 1 and Month 3	-1.000	0.317
		Month 1 and Month 4	-1.414	0.157
		Month 2 and Month 3	0.000	1.000
		Month 2 and Month 4	-1.414	0.157
		Month 3 and Month 4	-1.414	0.157
		Baseline vs Month 1	-1.414	0.157
		Baseline vs Month 2	0.000	1.000

	**3-4**	Baseline vs Month 3	-1.732	0.083
		Baseline vs Month 4	0.000	1.000
		Month 1 and Month 2	-1.414	0.157
		Month 1 and Month 3	-1.000	0.317
		Month 1 and Month 4	-1.414	0.157
		Month 2 and Month 3	-1.732	0.086
		Month 2 and Month 4	0.000	1.000
		Month 3 and Month 4	-1.732	0.083
	**>5**	Baseline vs Month 1	-1.732	0.083
		Baseline vs Month 2	-1.414	0.157
		Baseline vs Month 3	-2.449	**0.014**
		Baseline vs Month 4	-1.414	0.157
		Month 1 and Month 2	-1.000	0.317
		Month 1 and Month 3	-1.732	0.083
		Month 1 and Month 4	-1.000	0.317
		Month 2 and Month 3	-2.000	**0.046**
		Month 2 and Month 4	0.000	1.000
		Month 3 and Month 4	-2.000	**0.046**
**TRANSPORT (UP)**	**1-2**	Baseline vs Month 1	-1.732	0.083
		Baseline vs Month 2	-2.000	**0.046**
		Baseline vs Month 3	-1.000	0.317
		Baseline vs Month 4	-1.414	0.157
		Month 1 and Month 2	-1.000	0.317
		Month 1 and Month 3	-1.414	0.157
		Month 1 and Month 4	-1.000	0.317
		Month 2 and Month 3	-1.732	0.083
		Month 2 and Month 4	-1.414	0.157
		Month 3 and Month 4	-1.000	0.317
		Baseline vs Month 1	-1.000	0.317
		Baseline vs Month 2	-1.414	0.157

**IMPAIRED THOUGHT, CONCENTRATION, OR DIFFICULTY PROCESSING INFORMATION**	**MILD**	Baseline vs Month 3	-2.646	**0.008**
		Baseline vs Month 4	0.000	1.000
		Month 1 and Month 2	-1.000	0.317
		Month 1 and Month 3	-2.449	**0.014**
		Month 1 and Month 4	-1.000	0.317
		Month 2 and Month 3	-2.236	**0.025**
		Month 2 and Month 4	-1.414	0.157
		Month 3 and Month 4	-2.646	**0.008**
	**MODERATE**	Baseline vs Month 1	-1.732	0.083
		Baseline vs Month 2	-1.414	0.157
		Baseline vs Month 3	-1.414	0.157
		Baseline vs Month 4	-2.236	**0.025**
		Month 1 and Month 2	-1.000	0.317
		Month 1 and Month 3	-2.236	**0.025**
		Month 1 and Month 4	-1.414	0.157
		Month 2 and Month 3	-2.000	**0.046**
		Month 2 and Month 4	-1.732	0.083
		Month 3 and Month 4	-2.646	**0.008**
	**SEVERE**	Baseline vs Month 1	-1.414	0.157
		Baseline vs Month 2	0.000	1.000
		Baseline vs Month 3	-1.000	0.317
		Baseline vs Month 4	-1.732	0.083
		Month 1 and Month 2	-1.414	0.157
		Month 1 and Month 3	-1.732	0.083
		Month 1 and Month 4	-1.000	0.317
		Month 2 and Month 3	-1.000	0.317
		Month 2 and Month 4	-1.732	0.083
		Month 3 and Month 4	-2.000	**0.046**
		Baseline vs Month 1	-1.732	0.083
		Baseline vs Month 2	-1.000	0.317
		Baseline vs Month 3	-1.732	0.083

**SHORT OR LONG-TERM MEMORY LOSS**	**MODERATE**	Baseline vs Month 4	-1.000	0.317
		Month 1 and Month 2	-2.000	**0.046**
		Month 1 and Month 3	0.000	1.000
		Month 1 and Month 4	-1.414	0.157
		Month 2 and Month 3	-2.000	**0.046**
		Month 2 and Month 4	-1.414	0.157
		Month 3 and Month 4	-1.414	0.157
	**SEVERE**	Baseline vs Month 1	0.000	1.000
		Baseline vs Month 2	-1.000	0.317
		Baseline vs Month 3	0.000	1.000
		Baseline vs Month 4	-2.000	**0.046**
		Month 1 and Month 2	-1.000	0.317
		Month 1 and Month 3	0.000	1.000
		Month 1 and Month 4	-2.000	**0.046**
		Month 2 and Month 3	-1.000	0.317
		Month 2 and Month 4	-1.732	0.083
		Month 3 and Month 4	-2.000	**0.046**
**SLOWED SPEECH OR FREQUENTLY MIXING UP WORDS**	**MILD**	Baseline vs Month 1	-2.646	**0.008**
		Baseline vs Month 2	-2.449	**0.014**
		Baseline vs Month 3	-2.449	**0.014**
		Baseline vs Month 4	-3.000	**0.003**
		Month 1 and Month 2	-1.000	0.317
		Month 1 and Month 3	-1.000	0.317
		Month 1 and Month 4	-1.414	0.157
		Month 2 and Month 3	0.000	1.000
		Month 2 and Month 4	-1.732	0.083
		Month 3 and Month 4	-1.732	0.083
		Baseline vs Month 1	-2.236	**0.025**
		Baseline vs Month 2	-3.162	**0.002**
		Baseline vs Month 3	-2.646	**0.008**
		Baseline vs Month 4	-2.646	**0.008**

	**MODERATE**	Month 1 and Month 2	-2.236	**0.025**
		Month 1 and Month 3	-1.414	0.157
		Month 1 and Month 4	-1.414	0.157
		Month 2 and Month 3	-1.732	0.083
		Month 2 and Month 4	-1.732	0.083
		Month 3 and Month 4	0.000	1.000
	**SEVERE**	Baseline vs Month 1	0.000	1.000
		Baseline vs Month 2	-2.449	**0.014**
		Baseline vs Month 3	-1.000	0.317
		Baseline vs Month 4	0.000	1.000
		Month 1 and Month 2	-2.449	**0.014**
		Month 1 and Month 3	-1.000	0.317
		Month 1 and Month 4	0.000	1.000
		Month 2 and Month 3	-2.236	**0.025**
		Month 2 and Month 4	-2.449	**0.014**
		Month 3 and Month 4	-1.000	0.317
**HEADACHES**	**MILD**	Baseline vs Month 1	-1.732	0.083
		Baseline vs Month 2	-2.449	**0.014**
		Baseline vs Month 3	-2.000	**0.046**
		Baseline vs Month 4	-2.000	**0.046**
		Month 1 and Month 2	-1.732	0.083
		Month 1 and Month 3	-1.000	0.317
		Month 1 and Month 4	-1.000	0.317
		Month 2 and Month 3	-1.414	0.157
		Month 2 and Month 4	-1.414	0.157
		Month 3 and Month 4	0.000	1.000
		Baseline vs Month 1	-1.414	0.157
		Baseline vs Month 2	-1.414	0.157
		Baseline vs Month 3	-1.000	0.317
		Baseline vs Month 4	-1.414	0.157
		Month 1 and Month 2	-2.000	**0.046**

	**MODERATE**	Month 1 and Month 3	-1.732	0.083
		Month 1 and Month 4	0.000	1.000
		Month 2 and Month 3	-1.000	0.317
		Month 2 and Month 4	-2.000	**0.046**
		Month 3 and Month 4	-1.732	0.083
	**SEVERE**	Baseline vs Month 1	-1.732	0.083
		Baseline vs Month 2	0.000	1.000
		Baseline vs Month 3	-1.000	0.317
		Baseline vs Month 4	0.000	1.000
		Month 1 and Month 2	-1.732	0.083
		Month 1 and Month 3	-2.000	**0.046**
		Month 1 and Month 4	-1.732	0.083
		Month 2 and Month 3	-1.000	0.317
		Month 2 and Month 4	0.000	1.000
		Month 3 and Month 4	-1.000	0.317
**MUSCLE PAIN**	**MODERATE**	Baseline vs Month 1	-1.000	0.317
		Baseline vs Month 2	-2.236	**0.025**
		Baseline vs Month 3	0.000	1.000
		Baseline vs Month 4	-2.000	**0.046**
		Month 1 and Month 2	-2.449	**0.014**
		Month 1 and Month 3	-1.000	0.317
		Month 1 and Month 4	-2.236	**0.025**
		Month 2 and Month 3	-2.236	**0.025**
		Month 2 and Month 4	-1.000	0.317
		Month 3 and Month 4	-2.000	**0.046**
	**SEVERE**	Baseline vs Month 1	-1.000	0.317
		Baseline vs Month 2	-2.236	**0.025**
		Baseline vs Month 3	-1.414	0.157
		Baseline vs Month 4	-1.732	0.083
		Month 1 and Month 2	-2.000	**0.046**
		Month 1 and Month 3	-1.000	0.317

		Month 1 and Month 4	-1.414	0.157
		Month 2 and Month 3	-1.732	0.083
		Month 2 and Month 4	-1.414	0.157
		Month 3 and Month 4	-1.000	0.317
	**EXTREME**	Baseline vs Month 1	-1.000	0.317
		Baseline vs Month 2	-1.414	0.157
		Baseline vs Month 3	-1.000	0.317
		Baseline vs Month 4	-1.414	0.157
		Month 1 and Month 2	-1.000	0.317
		Month 1 and Month 3	0.000	1.000
		Month 1 and Month 4	-1.732	0.083
		Month 2 and Month 3	-1.000	0.317
		Month 2 and Month 4	-2.000	**0.046**
		Month 3 and Month 4	-1.732	0.083
**JOINT PAIN**	**MODERATE**	Baseline vs Month 1	-1.000	0.317
		Baseline vs Month 2	-1.000	0.317
		Baseline vs Month 3	-2.236	**0.025**
		Baseline vs Month 4	-1.732	0.083
		Month 1 and Month 2	0.000	1.000
		Month 1 and Month 3	-2.449	**0.014**
		Month 1 and Month 4	-2.000	**0.046**
		Month 2 and Month 3	-2.449	**0.014**
		Month 2 and Month 4	-2.000	**0.046**
		Month 3 and Month 4	-1.414	0.157
	**SEVERE**	Baseline vs Month 1	-1.000	0.317
		Baseline vs Month 2	-1.000	0.317
		Baseline vs Month 3	-2.236	**0.025**
		Baseline vs Month 4	-1.732	0.083
		Month 1 and Month 2	0.000	1.000
		Month 1 and Month 3	-2.449	**0.014**
		Month 1 and Month 4	-2.000	**0.046**

		Month 2 and Month 3	-2.449	**0.014**
		Month 2 and Month 4	-2.000	**0.046**
		Month 3 and Month 4	-1.414	0.157
	**EXTREME**	Baseline vs Month 1	0.000	1.000
		Baseline vs Month 2	-1.000	0.317
		Baseline vs Month 3	0.000	1.000
		Baseline vs Month 4	-1.000	0.317
		Month 1 and Month 2	-1.000	0.317
		Month 1 and Month 3	0.000	1.000
		Month 1 and Month 4	-1.000	0.317
		Month 2 and Month 3	-1.000	0.317
		Month 2 and Month 4	0.000	1.000
		Month 3 and Month 4	-1.000	0.317
**SLEEP DISTURBANCES**	**MILD**	Baseline vs Month 1	-1.732	0.083
		Baseline vs Month 2	-1.000	0.317
		Baseline vs Month 3	-1.000	0.317
		Baseline vs Month 4	-1.414	0.157
		Month 1 and Month 2	-1.414	0.157
		Month 1 and Month 3	-2.000	**0.046**
		Month 1 and Month 4	-2.236	**0.025**
		Month 2 and Month 3	-1.414	0.157
		Month 2 and Month 4	-1.732	0.083
		Month 3 and Month 4	-1.000	0.317
	**MODERATE**	Baseline vs Month 1	-1.732	0.083
		Baseline vs Month 2	-1.000	0.317
		Baseline vs Month 3	-1.000	0.317
		Baseline vs Month 4	-1.732	0.083
		Month 1 and Month 2	-1.414	0.157
		Month 1 and Month 3	-2.000	**0.046**
		Month 1 and Month 4	-2.449	**0.014**
		Month 2 and Month 3	-1.414	0.157

		Month 2 and Month 4	-2.000	**0.046**
		Month 3 and Month 4	-1.414	0.157
	**SEVERE**	Baseline vs Month 1	0.000	1.000
		Baseline vs Month 2	-1.000	0.317
		Baseline vs Month 3	-2.000	**0.046**
		Baseline vs Month 4	-2.000	**0.046**
		Month 1 and Month 2	-1.000	0.317
		Month 1 and Month 3	-2.000	**0.046**
		Month 1 and Month 4	-2.000	**0.046**
		Month 2 and Month 3	-1.732	0.083
		Month 2 and Month 4	-1.732	0.083
		Month 3 and Month 4	0.000	1.000
**SENSITIVITY TO LIGHT**	**MILD**	Baseline vs Month 1	-2.000	**0.046**
		Baseline vs Month 2	0.000	1.000
		Baseline vs Month 3	-1.000	0.317
		Baseline vs Month 4	-1.000	0.317
		Month 1 and Month 2	-2.000	**0.046**
		Month 1 and Month 3	-1.732	0.083
		Month 1 and Month 4	-1.732	0.083
		Month 2 and Month 3	-1.000	0.317
		Month 2 and Month 4	-1.000	0.317
		Month 3 and Month 4	0.000	1.000
	**MODERATE**	Baseline vs Month 1	0.000	1.000
		Baseline vs Month 2	-1.414	0.157
		Baseline vs Month 3	-1.000	0.317
		Baseline vs Month 4	-1.414	0.157
		Month 1 and Month 2	-1.414	0.157
		Month 1 and Month 3	-1.000	0.317
		Month 1 and Month 4	-1.414	0.157
		Month 2 and Month 3	-1.732	0.083
		Month 2 and Month 4	-2.000	**0.046**

		Month 3 and Month 4	-1.000	0.317
	SEVERE	Baseline vs Month 1	-1.414	0.157
		Baseline vs Month 2	-2.000	**0.046**
		Baseline vs Month 3	0.000	1.000
		Baseline vs Month 4	0.000	1.000
		Month 1 and Month 2	-2.449	**0.014**
		Month 1 and Month 3	-1.414	0.157
		Month 1 and Month 4	-1.414	0.157
		Month 2 and Month 3	-2.000	**0.046**
		Month 2 and Month 4	-2.000	**0.046**
		Month 3 and Month 4	0.000	1.000
**SENSITIVITY TO VIBRATION AND TOUCH**	**NONE**	Baseline vs Month 1	-1.732	0.083
		Baseline vs Month 2	-2.236	**0.025**
		Baseline vs Month 3	-2.000	**0.046**
		Baseline vs Month 4	-2.449	**0.014**
		Month 1 and Month 2	-1.414	0.157
		Month 1 and Month 3	-1.000	0.317
		Month 1 and Month 4	-1.732	0.083
		Month 2 and Month 3	-1.000	0.317
		Month 2 and Month 4	-1.000	0.317
		Month 3 and Month 4	-1.414	0.157
	**MILD**	Baseline vs Month 1	-2.236	**0.025**
		Baseline vs Month 2	-2.236	**0.025**
		Baseline vs Month 3	-2.236	**0.025**
		Baseline vs Month 4	-3.000	**0.003**
		Month 1 and Month 2	0.000	1.000
		Month 1 and Month 3	0.000	1.000
		Month 1 and Month 4	-2.000	**0.046**
		Month 2 and Month 3	0.000	1.000
		Month 2 and Month 4	-2.000	**0.046**
		Month 3 and Month 4	-2.000	**0.046**

	**MODERATE**	Baseline vs Month 1	-2.236	**0.025**
		Baseline vs Month 2	0.000	1.000
		Baseline vs Month 3	-1.732	0.083
		Baseline vs Month 4	-2.236	**0.025**
		Month 1 and Month 2	-2.236	**0.025**
		Month 1 and Month 3	-1.414	0.157
		Month 1 and Month 4	0.000	1.000
		Month 2 and Month 3	-1.732	0.083
		Month 2 and Month 4	-2.236	**0.025**
		Month 3 and Month 4	-1.414	0.157
**SENSITIVITY TO NOISE**	**MILD**	Baseline vs Month 1	-2.000	**0.046**
		Baseline vs Month 2	-2.000	**0.046**
		Baseline vs Month 3	-2.236	**0.025**
		Baseline vs Month 4	-1.414	0.157
		Month 1 and Month 2	0.000	1.000
		Month 1 and Month 3	-1.000	0.317
		Month 1 and Month 4	-1.414	0.157
		Month 2 and Month 3	-1.000	0.317
		Month 2 and Month 4	-1.414	0.157
		Month 3 and Month 4	-1.732	0.083
	**MODERATE**	Baseline vs Month 1	-2.000	**0.046**
		Baseline vs Month 2	-2.449	**0.014**
		Baseline vs Month 3	-2.828	**0.005**
		Baseline vs Month 4	-2.236	**0.025**
		Month 1 and Month 2	-1.414	0.157
		Month 1 and Month 3	-2.000	**0.046**
		Month 1 and Month 4	-1.000	0.317
		Month 2 and Month 3	-1.414	0.157
		Month 2 and Month 4	-1.000	0.317
		Month 3 and Month 4	-1.732	0.083
		Baseline vs Month 1	-2.000	**0.046**

**SENSITIVITY TO TASTE**	**NONE**	Baseline vs Month 2	-2.236	**0.025**
		Baseline vs Month 3	-1.414	0.157
		Baseline vs Month 4	-2.000	**0.046**
		Month 1 and Month 2	-1.000	0.317
		Month 1 and Month 3	-1.414	0.157
		Month 1 and Month 4	0.000	1.000
		Month 2 and Month 3	-1.732	0.083
		Month 2 and Month 4	-1.000	0.317
		Month 3 and Month 4	-1.414	0.157
	**MILD**	Baseline vs Month 1	-1.732	0.083
		Baseline vs Month 2	-2.000	**0.046**
		Baseline vs Month 3	-1.000	0.317
		Baseline vs Month 4	-1.000	0.317
		Month 1 and Month 2	-1.000	0.317
		Month 1 and Month 3	-2.000	**0.046**
		Month 1 and Month 4	-2.000	**0.046**
		Month 2 and Month 3	-2.236	**0.025**
		Month 2 and Month 4	-2.236	**0.025**
		Month 3 and Month 4	0.000	1.000
	**MODERATE**	Baseline vs Month 1	-1.414	0.157
		Baseline vs Month 2	0.000	1.000
		Baseline vs Month 3	-1.414	0.157
		Baseline vs Month 4	-2.000	**0.046**
		Month 1 and Month 2	-1.414	0.157
		Month 1 and Month 3	-2.000	**0.046**
		Month 1 and Month 4	-2.449	**0.014**
		Month 2 and Month 3	-1.414	0.157
		Month 2 and Month 4	-2.000	**0.046**
		Month 3 and Month 4	-1.414	0.157
		Baseline vs Month 1	-1.414	0.157
		Baseline vs Month 2	-1.732	0.083

SENSITIVITY TO ODOUR	NONE	Baseline vs Month 3	0.000	1.000
		Baseline vs Month 4	-1.000	0.317
		Month 1 and Month 2	-2.236	**0.025**
		Month 1 and Month 3	-1.414	0.157
		Month 1 and Month 4	-1.732	0.083
		Month 2 and Month 3	-1.732	0.083
		Month 2 and Month 4	-1.414	0.157
		Month 3 and Month 4	-1.000	0.317
	MILD	Baseline vs Month 1	-1.732	0.083
		Baseline vs Month 2	-1.000	0.317
		Baseline vs Month 3	-1.732	0.083
		Baseline vs Month 4	-1.732	0.083
		Month 1 and Month 2	-1.414	0.157
		Month 1 and Month 3	-2.449	**0.014**
		Month 1 and Month 4	0.000	1.000
		Month 2 and Month 3	-2.000	**0.046**
		Month 2 and Month 4	-1.414	0.157
		Month 3 and Month 4	-2.449	**0.014**
	MODERATE	Baseline vs Month 1	0.000	1.000
		Baseline vs Month 2	-1.000	0.317
		Baseline vs Month 3	-2.236	**0.025**
		Baseline vs Month 4	-2.000	**0.046**
		Month 1 and Month 2	-1.000	0.317
		Month 1 and Month 3	-2.236	**0.025**
		Month 1 and Month 4	-2.000	**0.046**
		Month 2 and Month 3	-2.449	**0.014**
		Month 2 and Month 4	-1.732	0.083
		Month 3 and Month 4	-3.000	**0.003**
		Baseline vs Month 1	-2.449	**0.014**
		Baseline vs Month 2	-2.000	**0.046**
		Baseline vs Month 3	-2.000	**0.046**

**MUSCLE WEAKNESS**	**MILD**	Baseline vs Month 4	-1.000	0.317
		Month 1 and Month 2	-1.414	0.157
		Month 1 and Month 3	-1.414	0.157
		Month 1 and Month 4	-2.236	**0.025**
		Month 2 and Month 3	0.000	1.000
		Month 2 and Month 4	-1.732	0.083
		Month 3 and Month 4	-1.732	0.083
	**MODERATE**	Baseline vs Month 1	-2.236	**0.025**
		Baseline vs Month 2	-2.000	**0.046**
		Baseline vs Month 3	-2.449	**0.014**
		Baseline vs Month 4	-1.414	0.157
		Month 1 and Month 2	-1.000	0.317
		Month 1 and Month 3	-1.000	0.317
		Month 1 and Month 4	-1.732	0.083
		Month 2 and Month 3	-1.414	0.157
		Month 2 and Month 4	-1.414	0.157
		Month 3 and Month 4	-2.000	**0.046**
**POOR COORDINATION**	**MILD**	Baseline vs Month 1	-1.000	0.317
		Baseline vs Month 2	0.000	1.000
		Baseline vs Month 3	-1.000	0.317
		Baseline vs Month 4	-2.000	**0.046**
		Month 1 and Month 2	-1.000	0.317
		Month 1 and Month 3	-1.414	0.157
		Month 1 and Month 4	-2.236	**0.025**
		Month 2 and Month 3	-1.000	0.317
		Month 2 and Month 4	-2.000	**0.046**
		Month 3 and Month 4	-1.732	0.083
		Baseline vs Month 1	0.000	1.000
		Baseline vs Month 2	-1.000	0.317
		Baseline vs Month 3	-1.000	0.317
		Baseline vs Month 4	-2.000	**0.046**

	**MODERATE**	Month 1 and Month 2	-1.000	0.317
		Month 1 and Month 3	-1.000	0.317
		Month 1 and Month 4	-2.000	**0.046**
		Month 2 and Month 3	0.000	1.000
		Month 2 and Month 4	-2.236	**0.025**
		Month 3 and Month 4	-2.236	**0.025**
**SORE THROAT**	**MILD**	Baseline vs Month 1	-2.236	**0.025**
		Baseline vs Month 2	-1.414	0.157
		Baseline vs Month 3	-2.000	**0.046**
		Baseline vs Month 4	-2.236	**0.025**
		Month 1 and Month 2	-1.732	0.083
		Month 1 and Month 3	-1.000	0.317
		Month 1 and Month 4	0.000	1.000
		Month 2 and Month 3	-1.414	0.157
		Month 2 and Month 4	-1.732	0.083
		Month 3 and Month 4	-1.000	0.317
	**MODERATE**	Baseline vs Month 1	-2.449	**0.014**
		Baseline vs Month 2	-1.732	0.083
		Baseline vs Month 3	-2.449	**0.014**
		Baseline vs Month 4	-2.646	**0.008**
		Month 1 and Month 2	-1.732	0.083
		Month 1 and Month 3	0.000	1.000
		Month 1 and Month 4	-1.000	0.317
		Month 2 and Month 3	-1.732	0.083
		Month 2 and Month 4	-2.000	**0.046**
		Month 3 and Month 4	-1.000	0.317
	**SEVERE**	Baseline vs Month 1	-1.000	0.317
		Baseline vs Month 2	-1.732	0.083
		Baseline vs Month 3	-1.732	0.083
		Baseline vs Month 4	-1.732	0.083
		Month 1 and Month 2	-1.414	0.157

		Month 1 and Month 3	-1.414	0.157
		Month 1 and Month 4	-1.414	0.157
		Month 2 and Month 3	0.000	1.000
		Month 2 and Month 4	0.000	1.000
		Month 3 and Month 4	0.000	1.000
**TENDER LYMPH NODES**	**NONE**	Baseline vs Month 1	-1.414	0.157
		Baseline vs Month 2	-1.000	0.317
		Baseline vs Month 3	-1.732	0.083
		Baseline vs Month 4	0.000	1.000
		Month 1 and Month 2	-1.732	0.083
		Month 1 and Month 3	-1.000	0.317
		Month 1 and Month 4	-1.414	0.157
		Month 2 and Month 3	-2.000	**0.046**
		Month 2 and Month 4	-1.000	0.317
		Month 3 and Month 4	-1.732	0.083
	**MILD**	Baseline vs Month 1	-1.000	0.317
		Baseline vs Month 2	-1.414	0.157
		Baseline vs Month 3	-2.000	**0.046**
		Baseline vs Month 4	-1.000	0.317
		Month 1 and Month 2	-1.732	0.083
		Month 1 and Month 3	-2.236	**0.025**
		Month 1 and Month 4	-1.414	0.157
		Month 2 and Month 3	-1.414	0.157
		Month 2 and Month 4	-1.000	0.317
		Month 3 and Month 4	-1.732	0.083
	**MODERATE**	Baseline vs Month 1	-2.000	**0.046**
		Baseline vs Month 2	-1.414	0.157
		Baseline vs Month 3	-1.414	0.157
		Baseline vs Month 4	-1.414	0.157
		Month 1 and Month 2	-2.449	**0.014**
		Month 1 and Month 3	-1.414	0.157

		Month 1 and Month 4	-1.414	0.157
		Month 2 and Month 3	-2.000	**0.046**
		Month 2 and Month 4	-2.000	**0.046**
		Month 3 and Month 4	0.000	1.000
**OTHER FLU-LIKE SYMPTOMS**	**MILD**	Baseline vs Month 1	-2.000	**0.046**
		Baseline vs Month 2	-1.000	0.317
		Baseline vs Month 3	-2.000	**0.046**
		Baseline vs Month 4	-2.000	**0.046**
		Month 1 and Month 2	-1.732	0.083
		Month 1 and Month 3	-2.828	**0.005**
		Month 1 and Month 4	0.000	1.000
		Month 2 and Month 3	-2.236	**0.025**
		Month 2 and Month 4	-1.732	0.083
		Month 3 and Month 4	-2.828	**0.005**
	**MODERATE**	Baseline vs Month 1	-1.732	0.083
		Baseline vs Month 2	-1.000	0.317
		Baseline vs Month 3	-1.732	0.083
		Baseline vs Month 4	-1.732	0.083
		Month 1 and Month 2	-2.000	**0.046**
		Month 1 and Month 3	-2.449	**0.014**
		Month 1 and Month 4	0.000	1.000
		Month 2 and Month 3	-1.414	0.157
		Month 2 and Month 4	-2.000	**0.046**
		Month 3 and Month 4	-2.449	**0.014**
	**NONE**	Baseline vs Month 1	0.000	1.000
		Baseline vs Month 2	-1.000	0.317
		Baseline vs Month 3	-1.414	0.157
		Baseline vs Month 4	-2.236	**0.025**
		Month 1 and Month 2	-1.000	0.317
		Month 1 and Month 3	-1.414	0.157
		Month 1 and Month 4	-2.236	**0.025**

PREDISPOSITION TO OTHER INFECTIONS		Month 2 and Month 3	-1.000	0.317
		Month 2 and Month 4	-2.000	**0.046**
		Month 3 and Month 4	-1.732	0.083
	MILD	Baseline vs Month 1	0.000	1.000
		Baseline vs Month 2	-1.732	0.083
		Baseline vs Month 3	-2.000	**0.046**
		Baseline vs Month 4	-2.646	**0.008**
		Month 1 and Month 2	-1.732	0.083
		Month 1 and Month 3	-2.000	**0.046**
		Month 1 and Month 4	-2.646	**0.008**
		Month 2 and Month 3	-1.000	0.317
		Month 2 and Month 4	-2.000	**0.046**
		Month 3 and Month 4	-1.732	0.083
	MODERATE	Baseline vs Month 1	-1.414	0.157
		Baseline vs Month 2	-2.000	**0.046**
		Baseline vs Month 3	-1.732	0.083
		Baseline vs Month 4	0.000	1.000
		Month 1 and Month 2	-1.414	0.157
		Month 1 and Month 3	-1.000	0.317
		Month 1 and Month 4	-1.414	0.157
		Month 2 and Month 3	-1.000	0.317
		Month 2 and Month 4	-2.000	**0.046**
		Month 3 and Month 4	-1.732	0.083
	SEVERE	Baseline vs Month 1	-1.414	0.157
		Baseline vs Month 2	-2.000	**0.046**
		Baseline vs Month 3	-1.732	0.083
		Baseline vs Month 4	-1.732	0.083
		Month 1 and Month 2	-1.414	0.157
		Month 1 and Month 3	-1.000	0.317
		Month 1 and Month 4	-1.000	0.317
		Month 2 and Month 3	-1.000	0.317

		Month 2 and Month 4	-1.000	0.317
		Month 3 and Month 4	0.000	1.000
**REACTED DIFFERENTLY TO COMMON ILLNESSES (e.g. common cold)**	**MILD**	Baseline vs Month 1	-1.000	0.317
		Baseline vs Month 2	-1.000	0.317
		Baseline vs Month 3	-2.449	**0.014**
		Baseline vs Month 4	-2.236	**0.025**
		Month 1 and Month 2	-1.414	0.157
		Month 1 and Month 3	-2.646	**0.008**
		Month 1 and Month 4	-2.449	**0.014**
		Month 2 and Month 3	-2.236	**0.025**
		Month 2 and Month 4	-2.000	**0.046**
		Month 3 and Month 4	-1.000	0.317
**NAUSEA**	**NONE**	Baseline vs Month 1	-1.000	0.317
		Baseline vs Month 2	-1.732	0.083
		Baseline vs Month 3	-1.732	0.083
		Baseline vs Month 4	-1.000	0.317
		Month 1 and Month 2	-2.000	**0.046**
		Month 1 and Month 3	-2.000	**0.046**
		Month 1 and Month 4	0.000	1.000
		Month 2 and Month 3	0.000	1.000
		Month 2 and Month 4	-2.000	**0.046**
		Month 3 and Month 4	-2.000	**0.046**
	**MILD**	Baseline vs Month 1	-1.732	0.083
		Baseline vs Month 2	-1.414	0.157
		Baseline vs Month 3	-2.000	**0.046**
		Baseline vs Month 4	-1.732	0.083
		Month 1 and Month 2	-2.236	**0.025**
		Month 1 and Month 3	-2.646	**0.008**
		Month 1 and Month 4	0.000	1.000
		Month 2 and Month 3	-1.414	0.157
		Month 2 and Month 4	-2.236	**0.025**

		Month 3 and Month 4	-2.646	**0.008**
	MODERATE	Baseline vs Month 1	-1.000	0.317
		Baseline vs Month 2	-1.414	0.157
		Baseline vs Month 3	-1.414	0.157
		Baseline vs Month 4	-1.414	0.157
		Month 1 and Month 2	-1.000	0.317
		Month 1 and Month 3	-1.732	0.083
		Month 1 and Month 4	-1.000	0.317
		Month 2 and Month 3	-2.000	**0.046**
		Month 2 and Month 4	0.000	1.000
		Month 3 and Month 4	-2.000	**0.046**
	SEVERE	Baseline vs Month 1	-1.414	0.157
		Baseline vs Month 2	-1.000	0.317
		Baseline vs Month 3	-1.414	0.157
		Baseline vs Month 4	-1.414	0.157
		Month 1 and Month 2	-1.732	0.083
		Month 1 and Month 3	0.000	1.000
		Month 1 and Month 4	0.000	1.000
		Month 2 and Month 3	-1.732	0.083
		Month 2 and Month 4	-1.732	0.083
		Month 3 and Month 4	0.000	1.000
ABDOMINAL PAIN	NONE	Baseline vs Month 1	-1.000	0.317
		Baseline vs Month 2	-1.000	0.317
		Baseline vs Month 3	-1.414	0.157
		Baseline vs Month 4	-1.732	0.083
		Month 1 and Month 2	-1.414	0.157
		Month 1 and Month 3	-1.732	0.083
		Month 1 and Month 4	-1.414	0.157
		Month 2 and Month 3	-1.000	0.317
		Month 2 and Month 4	-2.000	**0.046**
		Month 3 and Month 4	-2.236	**0.025**

	MILD	Baseline vs Month 1	0.000	1.000
		Baseline vs Month 2	-2.000	**0.046**
		Baseline vs Month 3	-2.449	**0.014**
		Baseline vs Month 4	-1.000	0.317
		Month 1 and Month 2	-2.000	**0.046**
		Month 1 and Month 3	-2.449	**0.014**
		Month 1 and Month 4	-1.000	0.317
		Month 2 and Month 3	-1.414	0.157
		Month 2 and Month 4	-2.236	**0.025**
		Month 3 and Month 4	-2.646	**0.008**
**IRRITABLE BOWEL**	**NONE**	Baseline vs Month 1	-2.000	**0.046**
		Baseline vs Month 2	-1.732	0.083
		Baseline vs Month 3	-2.000	**0.046**
		Baseline vs Month 4	-2.236	**0.025**
		Month 1 and Month 2	-1.000	0.317
		Month 1 and Month 3	0.000	1.000
		Month 1 and Month 4	-1.000	0.317
		Month 2 and Month 3	-1.000	0.317
		Month 2 and Month 4	-1.414	0.157
		Month 3 and Month 4	-1.000	0.317
	**MILD**	Baseline vs Month 1	-1.732	0.083
		Baseline vs Month 2	-1.414	0.157
		Baseline vs Month 3	-2.000	**0.046**
		Baseline vs Month 4	-1.732	0.083
		Month 1 and Month 2	-1.000	0.317
		Month 1 and Month 3	-1.000	0.317
		Month 1 and Month 4	0.000	1.000
		Month 2 and Month 3	-1.414	0.157
		Month 2 and Month 4	-1.000	0.317
		Month 3 and Month 4	-1.000	0.317
		Baseline vs Month 1	-1.000	0.317

	**MODERATE**	Baseline vs Month 2	-1.000	0.317
		Baseline vs Month 3	-1.000	0.317
		Baseline vs Month 4	-2.449	**0.014**
		Month 1 and Month 2	0.000	1.000
		Month 1 and Month 3	0.000	1.000
		Month 1 and Month 4	-2.236	**0.025**
		Month 2 and Month 3	0.000	1.000
		Month 2 and Month 4	-2.236	**0.025**
		Month 3 and Month 4	-2.236	**0.025**
**CHANGES IN FREQUENCY AND VOLUME OF URINATION**	**NONE**	Baseline vs Month 1	-2.000	**0.046**
		Baseline vs Month 2	-1.732	0.083
		Baseline vs Month 3	-1.732	0.083
		Baseline vs Month 4	-1.732	0.083
		Month 1 and Month 2	-1.000	0.317
		Month 1 and Month 3	-1.000	0.317
		Month 1 and Month 4	-1.000	0.317
		Month 2 and Month 3	0.000	1.000
		Month 2 and Month 4	0.000	1.000
		Month 3 and Month 4	0.000	1.000
	**MODERATE**	Baseline vs Month 1	-2.236	**0.025**
		Baseline vs Month 2	-2.000	**0.046**
		Baseline vs Month 3	-2.236	**0.025**
		Baseline vs Month 4	-2.449	**0.014**
		Month 1 and Month 2	-1.000	0.317
		Month 1 and Month 3	0.000	1.000
		Month 1 and Month 4	-1.000	0.317
		Month 2 and Month 3	-1.000	0.317
		Month 2 and Month 4	-1.414	0.157
		Month 3 and Month 4	-1.000	0.317
		Baseline vs Month 1	-1.000	0.317
		Baseline vs Month 2	-1.000	0.317

**SENSITIVITY TO FOOD, MEDICATIONS, OR CHEMICALS**	**MILD**	Baseline vs Month 3	-1.000	0.317
		Baseline vs Month 4	-1.732	0.083
		Month 1 and Month 2	-1.414	0.157
		Month 1 and Month 3	-1.414	0.157
		Month 1 and Month 4	-1.414	0.157
		Month 2 and Month 3	0.000	1.000
		Month 2 and Month 4	-2.000	**0.046**
		Month 3 and Month 4	-2.000	**0.046**
	**MODERATE**	Baseline vs Month 1	-1.000	0.317
		Baseline vs Month 2	-1.414	0.157
		Baseline vs Month 3	-1.414	0.157
		Baseline vs Month 4	-1.000	0.317
		Month 1 and Month 2	-1.732	0.083
		Month 1 and Month 3	-1.000	0.317
		Month 1 and Month 4	-1.414	0.157
		Month 2 and Month 3	-2.000	**0.046**
		Month 2 and Month 4	-1.000	0.317
		Month 3 and Month 4	-1.732	0.083
	**SEVERE**	Baseline vs Month 1	-1.000	0.317
		Baseline vs Month 2	-1.732	0.083
		Baseline vs Month 3	-1.000	0.317
		Baseline vs Month 4	-1.414	0.157
		Month 1 and Month 2	-2.000	**0.046**
		Month 1 and Month 3	-1.414	0.157
		Month 1 and Month 4	-1.732	0.083
		Month 2 and Month 3	-1.414	0.157
		Month 2 and Month 4	-1.000	0.317
		Month 3 and Month 4	-1.000	0.317
		Baseline vs Month 1	-1.000	0.317
		Baseline vs Month 2	-1.000	0.317
		Baseline vs Month 3	-2.000	**0.046**

**LIGHT-HEADEDNESS OR DIZZINESS**	**MILD**	Baseline vs Month 4	-1.732	0.083
		Month 1 and Month 2	0.000	1.000
		Month 1 and Month 3	-1.732	0.083
		Month 1 and Month 4	-1.414	0.157
		Month 2 and Month 3	-1.732	0.083
		Month 2 and Month 4	-1.414	0.157
		Month 3 and Month 4	-1.000	0.317
**ORTHOSTATIC INTOLERANCE (including POTS)**	**NONE**	Baseline vs Month 1	-1.414	0.157
		Baseline vs Month 2	-2.000	**0.046**
		Baseline vs Month 3	-1.414	0.157
		Baseline vs Month 4	-1.414	0.157
		Month 1 and Month 2	-1.414	0.157
		Month 1 and Month 3	0.000	1.000
		Month 1 and Month 4	0.000	1.000
		Month 2 and Month 3	-1.414	0.157
		Month 2 and Month 4	-1.414	0.157
		Month 3 and Month 4	0.000	1.000
	**MILD**	Baseline vs Month 1	-1.000	0.317
		Baseline vs Month 2	-1.000	0.317
		Baseline vs Month 3	-2.236	**0.025**
		Baseline vs Month 4	-2.236	**0.025**
		Month 1 and Month 2	0.000	1.000
		Month 1 and Month 3	-2.000	**0.046**
		Month 1 and Month 4	-2.000	**0.046**
		Month 2 and Month 3	-2.000	**0.046**
		Month 2 and Month 4	-2.000	**0.046**
		Month 3 and Month 4	0.000	1.000
		Baseline vs Month 1	-2.236	**0.025**
		Baseline vs Month 2	-2.000	**0.046**
		Baseline vs Month 3	-2.646	**0.008**
		Baseline vs Month 4	-2.000	**0.046**

	**MODERATE**	Month 1 and Month 2	-1.000	0.317
		Month 1 and Month 3	-1.414	0.157
		Month 1 and Month 4	-1.000	0.317
		Month 2 and Month 3	-1.732	0.083
		Month 2 and Month 4	0.000	1.000
		Month 3 and Month 4	-1.732	0.083
	**SEVERE**	Baseline vs Month 1	-1.732	0.083
		Baseline vs Month 2	-1.000	0.317
		Baseline vs Month 3	-1.000	0.317
		Baseline vs Month 4	-1.414	0.157
		Month 1 and Month 2	-2.000	**0.046**
		Month 1 and Month 3	-1.414	0.157
		Month 1 and Month 4	-2.236	**0.025**
		Month 2 and Month 3	-1.414	0.157
		Month 2 and Month 4	-1.000	0.317
		Month 3 and Month 4	-1.732	0.083
**ABNORMAL BODY TEMPERATURE**	**MILD**	Baseline vs Month 1	-2.000	**0.046**
		Baseline vs Month 2	-2.000	**0.046**
		Baseline vs Month 3	-2.236	**0.025**
		Baseline vs Month 4	-2.000	**0.046**
		Month 1 and Month 2	0.000	1.000
		Month 1 and Month 3	-1.000	0.317
		Month 1 and Month 4	0.000	1.000
		Month 2 and Month 3	-1.000	0.317
		Month 2 and Month 4	0.000	1.000
		Month 3 and Month 4	-1.000	0.317
		Baseline vs Month 1	0.000	1.000
		Baseline vs Month 2	-1.000	0.317
		Baseline vs Month 3	-1.732	0.083
		Baseline vs Month 4	-2.000	**0.046**
		Month 1 and Month 2	-1.000	0.317

	**MODERATE**	Month 1 and Month 3	-1.732	0.083
		Month 1 and Month 4	-2.000	**0.046**
		Month 2 and Month 3	-1.414	0.157
		Month 2 and Month 4	-1.732	0.083
		Month 3 and Month 4	-1.000	0.317
**COLDER EXTREMITIES**	**NONE**	Baseline vs Month 1	-1.732	0.083
		Baseline vs Month 2	0.000	1.000
		Baseline vs Month 3	-2.236	**0.025**
		Baseline vs Month 4	-2.646	**0.008**
		Month 1 and Month 2	-1.732	0.083
		Month 1 and Month 3	-1.414	0.157
		Month 1 and Month 4	-2.000	**0.046**
		Month 2 and Month 3	-2.236	**0.025**
		Month 2 and Month 4	-2.646	**0.008**
		Month 3 and Month 4	-1.414	0.157
	**MILD**	Baseline vs Month 1	-2.000	**0.046**
		Baseline vs Month 2	-1.000	0.317
		Baseline vs Month 3	-1.732	0.083
		Baseline vs Month 4	-2.236	**0.025**
		Month 1 and Month 2	-1.732	0.083
		Month 1 and Month 3	-1.000	0.317
		Month 1 and Month 4	-1.000	0.317
		Month 2 and Month 3	-1.414	0.157
		Month 2 and Month 4	-2.000	**0.046**
		Month 3 and Month 4	-1.414	0.157
	**MODERATE**	Baseline vs Month 1	-1.414	0.157
		Baseline vs Month 2	-2.000	**0.046**
		Baseline vs Month 3	-1.000	0.317
		Baseline vs Month 4	-1.000	0.317
		Month 1 and Month 2	-1.414	0.157
		Month 1 and Month 3	-1.732	0.083

		Month 1 and Month 4	-1.000	0.317
		Month 2 and Month 3	-2.236	**0.025**
		Month 2 and Month 4	-1.732	0.083
		Month 3 and Month 4	-1.414	0.157
	**SEVERE**	Baseline vs Month 1	-1.000	0.317
		Baseline vs Month 2	-1.732	0.083
		Baseline vs Month 3	-1.000	0.317
		Baseline vs Month 4	-2.000	**0.046**
		Month 1 and Month 2	-1.414	0.157
		Month 1 and Month 3	0.000	1.000
		Month 1 and Month 4	-1.732	0.083
		Month 2 and Month 3	-1.414	0.157
		Month 2 and Month 4	-1.000	0.317
		Month 3 and Month 4	-1.732	0.083
**INTOLERANCE TO EXTREME TEMPERATURES**	**NONE**	Baseline vs Month 1	-1.414	0.157
		Baseline vs Month 2	-1.000	0.317
		Baseline vs Month 3	-2.000	**0.046**
		Baseline vs Month 4	-2.449	**0.014**
		Month 1 and Month 2	-1.732	0.083
		Month 1 and Month 3	-1.414	0.157
		Month 1 and Month 4	-2.000	**0.046**
		Month 2 and Month 3	-2.236	**0.025**
		Month 2 and Month 4	-2.646	**0.008**
		Month 3 and Month 4	-1.414	0.157
	**MILD**	Baseline vs Month 1	-2.000	**0.046**
		Baseline vs Month 2	-1.000	0.317
		Baseline vs Month 3	-1.732	0.083
		Baseline vs Month 4	-2.236	**0.025**
		Month 1 and Month 2	-1.732	0.083
		Month 1 and Month 3	-1.000	0.317
		Month 1 and Month 4	-1.000	0.317

		Month 2 and Month 3	-1.414	0.157
		Month 2 and Month 4	-2.000	**0.046**
		Month 3 and Month 4	-1.414	0.157
	**MODERATE**	Baseline vs Month 1	-1.414	0.157
		Baseline vs Month 2	-2.236	**0.025**
		Baseline vs Month 3	-1.732	0.083
		Baseline vs Month 4	-1.000	0.317
		Month 1 and Month 2	-1.732	0.083
		Month 1 and Month 3	-1.000	0.317
		Month 1 and Month 4	-1.000	0.317
		Month 2 and Month 3	-1.414	0.157
		Month 2 and Month 4	-2.000	**0.046**
		Month 3 and Month 4	-1.414	0.157
	**SEVERE**	Baseline vs Month 1	-1.000	0.317
		Baseline vs Month 2	-2.000	**0.046**
		Baseline vs Month 3	-1.000	0.317
		Baseline vs Month 4	-2.000	**0.046**
		Month 1 and Month 2	-1.732	0.083
		Month 1 and Month 3	0.000	1.000
		Month 1 and Month 4	-1.732	0.083
		Month 2 and Month 3	-1.732	0.083
		Month 2 and Month 4	0.000	1.000
		Month 3 and Month 4	-1.732	0.083
	**MILD**	Baseline vs Month 1	-2.000	**0.046**
		Baseline vs Month 2	-1.000	0.317
		Baseline vs Month 3	-1.000	0.317
		Baseline vs Month 4	-1.414	0.157
		Month 1 and Month 2	-1.732	0.083
		Month 1 and Month 3	-1.732	0.083
		Month 1 and Month 4	-1.414	0.157
		Month 2 and Month 3	0.000	1.000

**SWEATING EPISODES**		Month 2 and Month 4	-1.000	0.317
		Month 3 and Month 4	-1.000	0.317
	**MODERATE**	Baseline vs Month 1	-1.414	0.157
		Baseline vs Month 2	-2.000	**0.046**
		Baseline vs Month 3	-1.000	0.317
		Baseline vs Month 4	-1.000	0.317
		Month 1 and Month 2	-1.414	0.157
		Month 1 and Month 3	-1.732	0.083
		Month 1 and Month 4	-1.000	0.317
		Month 2 and Month 3	-2.236	**0.025**
		Month 2 and Month 4	-1.732	0.083
		Month 3 and Month 4	-1.414	0.157
	**SEVERE**	Baseline vs Month 1	-2.000	**0.046**
		Baseline vs Month 2	-1.414	0.157
		Baseline vs Month 3	-1.414	0.157
		Baseline vs Month 4	-1.414	0.157
		Month 1 and Month 2	-1.414	0.157
		Month 1 and Month 3	-1.414	0.157
		Month 1 and Month 4	-1.414	0.157
		Month 2 and Month 3	0.000	1.000
		Month 2 and Month 4	0.000	1.000
		Month 3 and Month 4	0.000	1.000
