# Supplementary material for: Impact of Life Stressors on Myalgic Encephalomyelitis/Chronic Fatigue Syndrome Symptoms: An Australian Longitudinal Study
Source: Int J Environ Res Public Health. 2021 Oct 11;18(20):10614. doi: 10.3390/ijerph182010614 (PMC8535742; doi:10.3390/ijerph182010614)
Supplement: Supplementary file 1 [file ijerph-18-10614-s001.zip › Table S9. Partial correlation table.pdf]

Table S9. Partial correlation table

CORRELATIONS BETWEEN VARIABLES		CORRELATION COEFFICIENT	P value
WEEKLY WORK HOURS	ACCESS TO HEALTHCARE SERVICES		
	Occupational Therapy	-0.159	0.036
	PROFESSIONAL AND UNPAID SUPPORT		
	Meals (P)	-0.209	0.006
	Transport (P)	-0.190	0.012
	Household Chores (UP)	-0.245	0.001
	Meals (UP)	-0.193	0.011
	Transport (UP)	-0.157	0.039
	ME/CFS CLINICAL SYMPTOMS		
	Memory Loss	-0.172	0.024
	Sleep Disturbances	-0.183	0.016
	Muscle Weakness	-0.175	0.021
	Changes in frequency and volume of urination	-0.175	0.021
	Orthostatic Intolerance	-0.180	0.018
	Intolerance to extreme temperatures	-0.189	0.013
CHANGE IN WEEKLY WORK HOURS	FAMILY, FINANCIAL AND WORK-RELATED PARAMETERS		
	Sustainability of Household Income	0.253	0.001
	Change in Household Income	0.356	0.000
	PROFESSIONAL AND UNPAID SUPPORT		
	Meals (UP)	-0.205	0.007
	ME/CFS CLINICAL SYMPTOMS		
	Impaired Concentration	-0.165	0.030
	Muscle Pain	-0.188	0.013
	Muscle Weakness	-0.186	0.015
	Orthostatic Intolerance	-0.177	0.020

**SUSTAINABILTY OF HOUSEHOLD INCOME**	**FAMILY, FINANCIAL AND WORK-RELATED PARAMETERS**		
	**Change in Household Income**	0.188	0.013
	**PROFESSIONAL AND UNPAID SUPPORT**		
	**Childcare (P)**	-0.159	0.037
	**Household Chores (P)**	-0.218	0.004
	**Meals (P)**	-0.157	0.039
	**ME/CFS CLINICAL SYMPTOMS**		
	**Impaired Concentration**	-0.273	0.000
	**Muscle Pain**	-0.303	0.000
	**Joint Pain**	-0.199	0.009
	**Sleep Disturbances**	-0.283	0.000
	**Muscle Weakness**	-0.284	0.000
	**Nausea**	-0.154	0.043
	**Changes in frequency and volume of urination**	-0.244	0.001
	**Orthostatic Intolerance**	-0.244	0.001
	**Intolerance to extreme temperatures**	-0.174	0.022
**CHANGE IN HOUSEHOLD INCOME**	**FAMILY, FINANCIAL AND WORK-RELATED PARAMETERS**		
	**Living Arrangement**	-0.206	0.007
	**PROFESSIONAL AND UNPAID SUPPORT**		
	**Childcare (P)**	-0.221	0.004
	**Transport (P)**	0.215	0.005
	**Childcare (UP)**	-0.203	0.007
	**PROFESSIONAL AND UNPAID SUPPORT**		
	**Other Services (P)**	-0.199	0.009
	**Childcare (P)**	0.328	0.000
	**Childcare (UP)**	0.499	0.000
	**Household Chores (UP)**	-0.197	0.009
	**Personal Support (UP)**	-0.259	0.001

**LIVING ARRANGEMENT**	**Meals (UP)**	-0.155	0.042
	**Transport (UP)**	-0.207	0.006
	**ME/CFS CLINICAL SYMPTOMS**		
	**Headaches**	0.192	0.011
	**Joint Pain**	-0.196	0.010
	**Tender lymph nodes**	0.151	0.048
**GP**	**ACCESS TO HEALTHCARE SERVICES**		
	**Nurse**	0.196	0.010
	**Pathologist**	0.368	0.000
	**Medical Specialist**	0.343	0.000
	**Occupational Therapist**	0.245	0.001
	**Physiotherapist**	0.252	0.001
	**Other**	-0.154	0.043
	**PROFESSIONAL AND UNPAID SUPPORT**		
	**Household Chores (P)**	0.171	0.024
	**Meals (P)**	0.181	0.017
	**Transport (P)**	0.153	0.044
	**ME/CFS CLINICAL SYMPTOMS**		
	**Impaired Concentration**	0.157	0.039
	**Memory Loss**	0.193	0.011
	**Headaches**	0.183	0.016
	**Tender lymph nodes**	0.178	0.019
	**Abdominal Pain**	0.203	0.008
	**Irritable Bowel**	0.245	0.001
	**Changes in frequency and volume of urination**	0.191	0.012
	**Intolerance to extreme temperature**	0.151	0.047
	**ACCESS TO HEALTHCARE SERVICES**		
	**Pathologist**	0.288	0.000
	**Medical Specialist**	0.481	0.000

**NURSE**	**PROFESSIONAL AND UNPAID SUPPORT**		
	**Meals (P)**	0.178	0.019
	**Personal Support (UP)**	0.218	0.004
	**ME/CFS CLINICAL SYMPTOMS**		
	**Headaches**	0.207	0.006
	**Sleep Disturbances**	0.169	0.026
	**Sore Throat**	-0.156	0.041
	**Nausea**	0.149	0.050
**PATHOLOGIST**	**ACCESS TO HEALTHCARE SERVICES**		
	**Medical Specialist**	0.553	0.000
	**Occupational Therapist**	0.382	0.000
	**PROFESSIONAL AND UNPAID SUPPORT**		
	**Childcare (UP)**	0.243	0.001
	**ME/CFS CLINICAL SYMPTOMS**		
	**Headaches**	0.173	0.023
**MEDICAL SPECIALIST**	**ACCESS TO HEALTHCARE SERVICES**		
	**Occupational Therapist**	0.357	0.000
	**PROFESSIONAL AND UNPAID SUPPORT**		
	**Household Chores (P)**	0.196	0.010
	**Personal Support (P)**	0.152	0.046
	**Childcare (UP)**	0.178	0.019
	**Personal Support (UP)**	0.203	0.008
	**ME/CFS CLINICAL SYMPTOMS**		
	**Impaired Concentration**	0.185	0.016
	**Memory Loss**	0.229	0.003
	**Headaches**	0.238	0.002
	**Abdominal Pain**	0.177	0.022
	**ACCESS TO HEALTHCARE SERVICES**		

OCCUPATIONAL THERAPIST	**Physiotherapist**	0.266	0.000
	**PROFESSIONAL AND UNPAID SUPPORT**		
	**Household Chores (P)**	0.398	0.000
	**Personal Support (P)**	0.464	0.000
	**Meals (P)**	0.342	0.000
	**Transport (P)**	0.493	0.000
	**Childcare (UP)**	0.217	0.004
	**Meals (UP)**	-0.161	0.034
	**ME/CFS CLINICAL SYMPTOMS**		
	**Headaches**	0.162	0.033
	**Muscle Pain**	0.165	0.030
	**Muscle Weakness**	0.219	0.004
PHYSIOTHERAPIST	**PROFESSIONAL AND UNPAID SUPPORT**		
	**Childcare (P)**	0.169	0.026
	**Household Chores (P)**	0.235	0.002
	**Personal Support (P)**	0.206	0.007
	**Meals (P)**	0.281	0.000
	**Transport (P)**	0.245	0.001
	**Personal Support (UP)**	0.239	0.002
	**ME/CFS CLINICAL SYMPTOMS**		
	**Muscle Pain**	0.152	0.046
	**Sleep Disturbances**	0.260	0.001
OTHER HEALTHCARE SERVICE	**ME/CFS CLINICAL SYMPTOMS**		
	**Muscle Pain**	0.154	0.043
	**Joint Pain**	0.179	0.019
	**PROFESSIONAL AND UNPAID SUPPORT**		

**CHILDCARE (P)**	**Household Chores (P)**	0.228	0.003
	**Household Chores (UP)**	0.511	0.000
	**Personal Support (UP)**	-0.196	0.010
	**ME/CFS CLINICAL SYMPTOMS**		
	**Impaired Concentration**	0.190	0.013
	**Memory Loss**	0.201	0.008
	**Headaches**	0.247	0.001
	**Muscle Pain**	0.222	0.003
	**Sleep Disturbances**	0.170	0.026
	**Poor Coordination**	0.156	0.040
	**Tender Lymph Nodes**	0.207	0.006
	**Nausea**	0.176	0.021
	**Changes in frequency and volume of urination**	0.257	0.001
	**Intolerance to extreme temperatures**	0.160	0.035
	**PROFESSIONAL AND UNPAID SUPPORT**		
	**Personal Support (P)**	0.819	0.000
	**Meals (P)**	0.708	0.000
	**Transport (P)**	0.342	0.000
	**Childcare (UP)**	0.177	0.020
	**Meals (UP)**	0.238	0.002
	**ME/CFS CLINICAL SYMPTOMS**		
	**Impaired Concentration**	0.168	0.027
	**Muscle Pain**	0.455	0.000
	**Sleep Disturbances**	0.254	0.001
	**Sensitivity to Vibration and Touch**	0.231	0.002
	**Sensitivity to Taste**	0.397	0.000
	**Muscle Weakness**	0.398	0.000
	**Nausea**	0.369	0.000
	**Abdominal Pain**	0.161	0.035
	**Changes in frequency and volume of urination**	0.265	0.000

**HOUSEHOLD CHORES (P)**	**Orthostatic Intolerance**	0.352	0.000
	**Intolerance to extreme temperatures**	0.240	0.001
**PERSONAL SUPPORT (P)**	**PROFESSIONAL AND UNPAID SUPPORT**		
	**Meals (P)**	0.728	0.000
	**Transport (P)**	0.552	0.000
	**Household Chores (UP)**	-0.199	0.009
	**Transport (UP)**	-0.210	0.006
	**ME/CFS CLINICAL SYMPTOMS**		
	**Muscle Pain**	0.436	0.000
	**Sleep Disturbances**	0.227	0.003
	**Muscle Weakness**	0.348	0.000
	**Nausea**	0.273	0.000
	**Changes in frequency and volume of urination**	0.239	0.002
	**Orthostatic Intolerance**	0.424	0.000
	**Intolerance to extreme temperatures**	0.229	0.002
**MEALS (P)**	**PROFESSIONAL AND UNPAID SUPPORT**		
	**Transport (P)**	0.507	0.000
	**Personal Support (UP)**	0.235	0.002
	**Meals (UP)**	0.248	0.001
	**ME/CFS CLINICAL SYMPTOMS**		
	**Muscle Pain**	0.390	0.000
	**Joint Pain**	0.192	0.011
	**Sleep Disturbances**	0.413	0.000
	**Muscle Weakness**	0.403	0.000
	**Nausea**	0.336	0.000
	**Changes in frequency and volume of urination**	0.164	0.031
	**Orthostatic Intolerance**	0.405	0.000
	**Abnormal Body Temperature**	0.159	0.036

TRANSPORT (P)	PROFESSIONAL AND UNPAID SUPPORT		
	Transport (UP)	-0.159	0.037
	ME/CFS CLINICAL SYMPTOMS		
	Muscle Pain	0.267	0.000
	Joint Pain	0.201	0.008
	Sleep Disturbances	0.168	0.027
	Muscle Weakness	0.282	0.000
	Tender Lymph Nodes	0.210	0.006
	Orthostatic Intolerance	0.292	0.000
	Intolerance to extreme temperatures	0.357	0.000
CHILDCARE (UP)	ME/CFS CLINICAL SYMPTOMS		
	Impaired Concentration	0.159	0.036
	Memory Loss	0.181	0.017
	Headaches	0.216	0.004
	Muscle Weakness	0.158	0.038
	Changes in frequency and volume of urination	0.205	0.007
HOUSEHOLD CHORES (UP)	PROFESSIONAL AND UNPAID SUPPORT		
	Personal Support (UP)	0.403	0.000
	Meals (UP)	0.609	0.000
	Transport (UP)	0.600	0.000
	ME/CFS CLINICAL SYMPTOMS		
	Impaired Concentration	0.194	0.011
	Memory Loss	0.349	0.000
	Muscle Weakness	0.283	0.000
	Nausea	0.184	0.015
	Changes in frequency and volume of urination	0.171	0.025
	PROFESSIONAL AND UNPAID SUPPORT		

**PERSONAL SUPPORT (UP)**	**Meals (UP)**	0.499	0.000
	**Transport (UP)**	0.432	0.000
	**ME/CFS CLINICAL SYMPTOMS**		
	**Impaired Concentration**	0.199	0.009
	**Memory Loss**	0.266	0.000
	**Sleep Disturbances**	0.236	0.002
	**Muscle Weakness**	0.274	0.000
	**Nausea**	0.381	0.000
	**Abdominal Pain**	0.338	0.000
	**Changes in frequency and volume of urination**	0.162	0.034
**MEALS (UP)**	**PROFESSIONAL AND UNPAID SUPPORT**		
	**Transport (UP)**	0.630	0.000
	**ME/CFS CLINICAL SYMPTOMS**		
	**Impaired Concentration**	0.182	0.017
	**Memory Loss**	0.285	0.000
	**Headaches**	0.305	0.000
	**Sleep Disturbances**	0.201	0.008
	**Muscle Weakness**	0.380	0.000
	**Nausea**	0.424	0.000
	**Abdominal Pain**	0.201	0.008
	**Changes in frequency and volume of urination**	0.209	0.006
**TRANSPORT (UP)**	**ME/CFS CLINICAL SYMPTOMS**		
	**Memory Loss**	0.295	0.000
	**Nausea**	0.205	0.007
	**Abdominal Pain**	0.169	0.026
	**ME/CFS CLINICAL SYMPTOMS**		
	**Memory Loss**	0.731	0.000
	**Muscle Pain**	0.184	0.015

**IMPAIRED CONCENTRATION**	**Headaches**	0.445	0.000
	**Muscle Pain**	0.414	0.000
	**Joint Pain**	0.394	0.000
	**Sleep Disturbances**	0.210	0.006
	**Muscle Weakness**	0.542	0.000
	**Tender Lymph Nodes**	0.241	0.001
	**Nausea**	0.316	0.000
	**Abdominal Pain**	0.315	0.000
	**Changes in frequency and volume of urination**	0.244	0.001
	**Orthostatic Intolerance**	0.298	0.000
	**Intolerance to extreme temperatures**	0.317	0.000
**MEMORY LOSS**	**ME/CFS CLINICAL SYMPTOMS**		
	**Headaches**	0.285	0.000
	**Muscle Pain**	0.367	0.000
	**Joint Pain**	0.363	0.000
	**Sleep Disturbances**	0.362	0.000
	**Muscle Weakness**	0.493	0.000
	**Nausea**	0.365	0.000
	**Abdominal Pain**	0.314	0.000
	**Changes in frequency and volume of urination**	0.479	0.000
	**Orthostatic Intolerance**	0.214	0.005
	**Abnormal body temperature**	0.176	0.021
	**Intolerance to extreme temperatures**	0.187	0.014
**HEADACHES**	**ME/CFS CLINICAL SYMPTOMS**		
	**Sleep Disturbances**	0.159	0.036
	**Tender Lymph Nodes**	0.232	0.002
	**Nausea**	0.184	0.015
	**Abdominal Pain**	0.339	0.000
	**Changes in frequency and volume of urination**	0.405	0.000

	**Intolerance to extreme temperatures**	0.191	0.011
**MUSCLE PAIN**	**ME/CFS CLINICAL SYMPTOMS**		
	**Joint Pain**	0.538	0.000
	**Sleep Disturbances**	0.542	0.000
	**Muscle Weakness**	0.603	0.000
	**Tender Lymph Nodes**	0.176	0.020
	**Nausea**	0.392	0.000
	**Abdominal Pain**	0.224	0.003
	**Changes in frequency and volume of urination**	0.340	0.000
	**Orthostatic Intolerance**	0.480	0.000
	**Intolerance to extreme temperatures**	0.259	0.001
**JOINT PAIN**	**ME/CFS CLINICAL SYMPTOMS**		
	**Sleep Disturbances**	0.428	0.000
	**Muscle Weakness**	0.305	0.000
	**Tender Lymph Nodes**	0.252	0.001
	**Nausea**	0.308	0.000
	**Abdominal Pain**	0.207	0.006
	**Changes in frequency and volume of urination**	0.317	0.000
	**Orthostatic Intolerance**	0.440	0.000
	**Intolerance to extreme temperatures**	0.360	0.000
**MUSCLE WEAKNESS**	**ME/CFS CLINICAL SYMPTOMS**		
	**Tender Lymph Nodes**	0.155	0.042
	**Nausea**	0.442	0.000
	**Abdominal Pain**	0.192	0.011
	**Changes in frequency and volume of urination**	0.409	0.000
	**Dizziness**	0.541	0.000
	**Orthostatic Intolerance**	0.386	0.000
	**Intolerance to extreme temperatures**	0.315	0.000

**SORE THROAT**	**ME/CFS CLINICAL SYMPTOMS**		
	**Tender Lymph Nodes**	0.569	0.000
	**Orthostatic Intolerance**	0.266	0.000
	**Abnormal body temperature**	0.308	0.000
	**Intolerance to extreme temperatures**	0.190	0.012
**TENDER LYMPH NODES**	**ME/CFS CLINICAL SYMPTOMS**		
	**Changes in frequency and volume of urination**	0.330	0.000
	**Orthostatic Intolerance**	0.252	0.001
	**Intolerance to extreme temperatures**	0.375	0.000
**NAUSEA**	**ME/CFS CLINICAL SYMPTOMS**		
	**Abdominal Pain**	0.503	0.000
	**Irritable Bowel**	0.460	0.000
	**Changes in frequency and volume of urination**	0.488	0.000
	**Sensitivity to food, medications, or chemicals**	0.181	0.017
	**Dizziness**	0.537	0.000
	**Orthostatic Intolerance**	0.471	0.000
	**Abnormal body temperature**	0.320	0.000
	**Colder extremities**	0.318	0.000
	**Intolerance to extreme temperatures**	0.446	0.000
	**Sweating Episodes**	0.205	0.007
**ABDOMINAL PAIN**	**ME/CFS CLINICAL SYMPTOMS**		
	**Irritable Bowel**	0.523	0.000
	**Changes in frequency and volume of urination**	0.294	0.000
	**Sensitivity to food, medications, or chemicals**	0.230	0.002
	**Dizziness**	0.438	0.000
	**Orthostatic Intolerance**	0.346	0.000
	**Abnormal body temperature**	0.301	0.000

	**Colder extremities**	0.376	0.000
	**Intolerance to extreme temperatures**	0.338	0.000
**CHANGES IN FREQUENCY AND VOLUME OF URINATION**	**ME/CFS CLINICAL SYMPTOMS**		
	**Sensitivity to food, medications, or chemicals**	0.502	0.000
	**Dizziness**	0.423	0.001
	**Orthostatic Intolerance**	0.448	0.000
	**Abnormal body temperature**	0.402	0.000
	**Colder extremities**	0.386	0.000
	**Intolerance to extreme temperatures**	0.483	0.000
**SENSITIVITY TO FOOD, MEDICATIONS, OR CHEMICALS**	**ME/CFS CLINICAL SYMPTOMS**		
	**Orthostatic Intolerance**	0.238	0.002
	**Abnormal body temperature**	0.325	0.000
	**Colder extremities**	0.255	0.001
	**Intolerance to extreme temperatures**	0.400	0.000
**ORTHOSTATIC INTOLERANCE**	**ME/CFS CLINICAL SYMPTOMS**		
	**Intolerance to extreme temperatures**	0.572	0.000
